# Development of the First Tritiated Tetrazine: Facilitating Tritiation of Proteins

**DOI:** 10.1002/cbic.202200539

**Published:** 2022-11-04

**Authors:** Natasha Shalina Radjani Bidesi, Umberto Maria Battisti, Sara Lopes van de Broek, Vladimir Shalgunov, Anne‐Mette Dall, Jesper Bøggild Kristensen, Dag Sehlin, Stina Syvänen, Gitte Moos Knudsen, Matthias Manfred Herth

**Affiliations:** ^1^ Department of Drug Design and Pharmacology Faculty of Health and Medical Sciences University of Copenhagen Jagtvej 160 2100 Copenhagen Denmark; ^2^ Department of Clinical Physiology Nuclear Medicine and PET Rigshospitalet Blegdamsvej 9 2100 Copenhagen Denmark; ^3^ Novo Nordisk A/S Smørmosevej 17–19, Bagsværd 2880 Copenhagen Denmark; ^4^ Rudbeck Laboratory, Department of Public Health and Caring Sciences Uppsala University Dag Hammarskjölds väg 20 75185 Uppsala Sweden; ^5^ Neurobiology Research Unit Rigshospitalet Blegdamsvej 9 2100 Copenhagen Denmark

**Keywords:** autoradiography, bioorthogonal chemistry, pre-targeting, tetrazine ligation, tritiation

## Abstract

Tetrazine (Tz)–*trans*‐cyclooctene (TCO) ligation is an ultra‐fast and highly selective reaction and it is particularly suited to label biomolecules under physiological conditions. As such, a ^3^H‐Tz based synthon would have wide applications for in vitro/ex vivo assays. In this study, we developed a ^3^H‐labeled Tz and characterized its potential for application to pretargeted autoradiography. Several strategies were explored to synthesize such a Tz. However, classical approaches such as reductive halogenation failed. For this reason, we designed a Tz containing an aldehyde and explored the possibility of reducing this group with NaBT_4_. This approach was successful and resulted in [^3^H]‐(4‐(6‐(pyridin‐2‐yl)‐1,2,4,5‐tetrazin‐3‐yl)phenyl)methan‐t‐ol with a radiochemical yield of 22 %, a radiochemical purity of 96 % and a molar activity of 0.437 GBq/μmol (11.8 Ci/mmol). The compound was successfully applied to pretargeted autoradiography. Thus, we report the synthesis of the first ^3^H‐labeled Tz and its successful application as a labeling building block.

## Graphical abstract

**A tritiated tetrazine** based on a phenyl/pyridyl core structure was successfully radiolabeled with NaBT_4_ by reducing an aldehyde moiety. The synthon was effectively employed to label an antibody in vitro with pretargeted autoradiography.

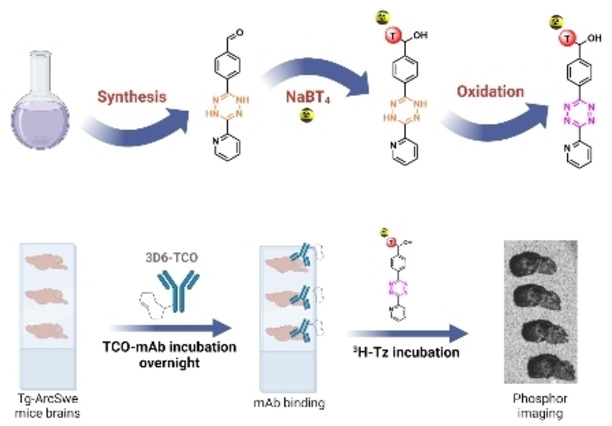

## 1 Introduction

**Figure 1:**
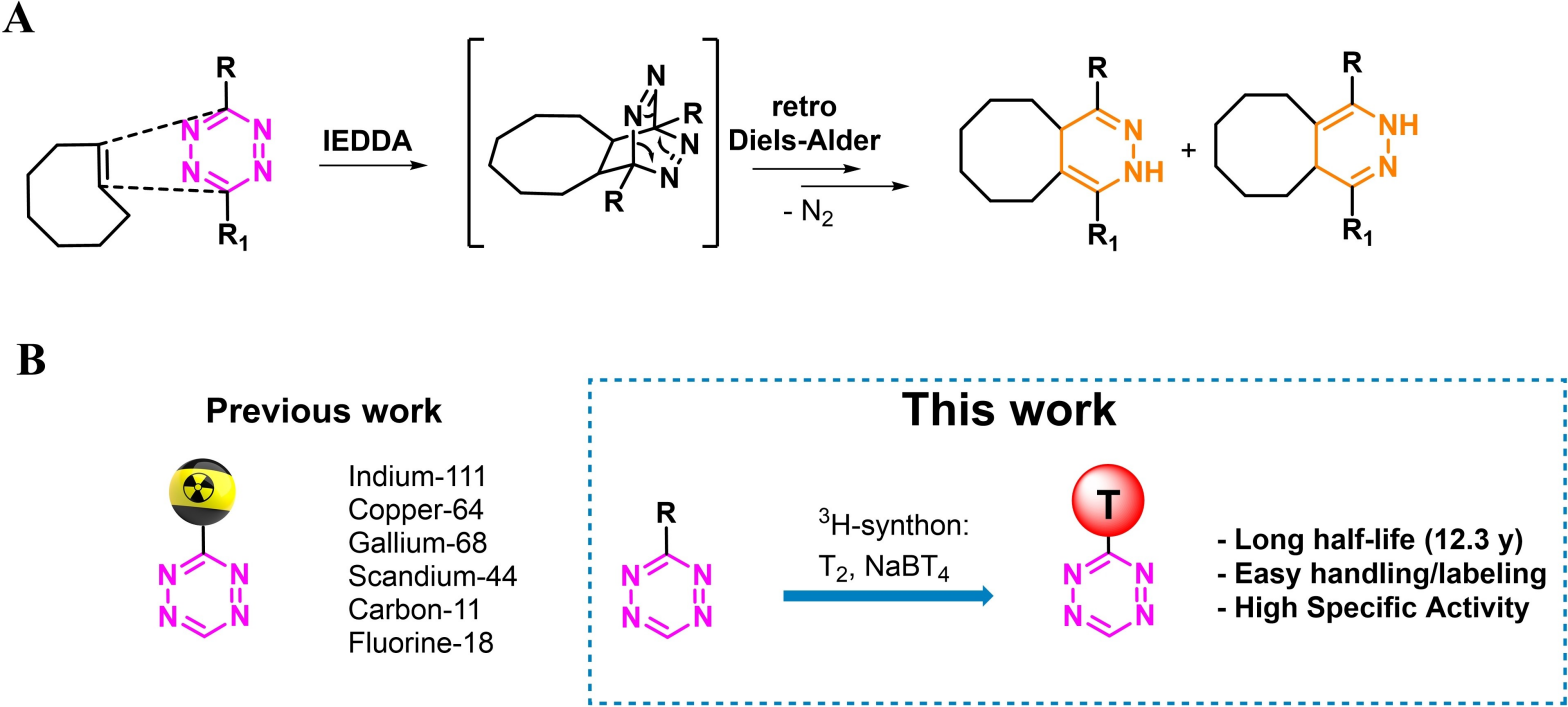
A) The tetrazine‐TCO ligation. B) Previously reported tetrazines were labeled with fluorine‐18, carbon‐11, copper‐64, gallium‐68, scandium‐44 (pet) and indium‐111 (SPECT) radionuclides. This study reports the first synthesis of a ^3^H‐labeled Tz.

Recently, the interest in tetrazines (Tz) has exponentially increased due to the emergence of click‐chemistry as an important tool in drug development. These molecules are usually employed as bioorthogonal reagents with strained alkenes (e. g. *trans*‐cyclooctenes (TCOs)) in a reaction called tetrazine ligation. The Tz ligation occurs by means of the inverse electron‐demand Diels‐Alder (iEDDA) cycloaddition, followed by the retro‐Diels Alder resulting in N_2_ elimination (Figure 1A).^[1]^ The wide range of applications of this transformation includes but are not limited to: live cell imaging, bioconjugation, nanoscience and nuclear medicine, the latter both for therapeutic and diagnostic (even theranostic) applications.[^1c^, ^2^] Moreover, the TCO‐Tz ligation holds favorable properties such as high selectivity and superior reaction kinetics rate constants (up to 10^6−7^ M^−1^ s^−1^) compared to other (bio)orthogonal tools.^[3]^ Over the years, several Tzs radiolabeling strategies emerged employing different nuclides such as fluorine‐18, carbon‐11, iodine‐123 or indium‐111 (Figure 1B).^[4]^ However, to the best of our knowledge, the synthesis of a tritiated Tz has never been reported. Tritium labeled compounds are essential to drug discovery due to their relatively ease and safety to handle.^[5]^ In this study, we report the first synthesis of a ^3^H‐labeled Tz as well as its application for pretargeted autoradiography (Figure 1B).^[6]^

## 2 Results and Discussion

### 2.1 Initial attempts

**Scheme 1:**
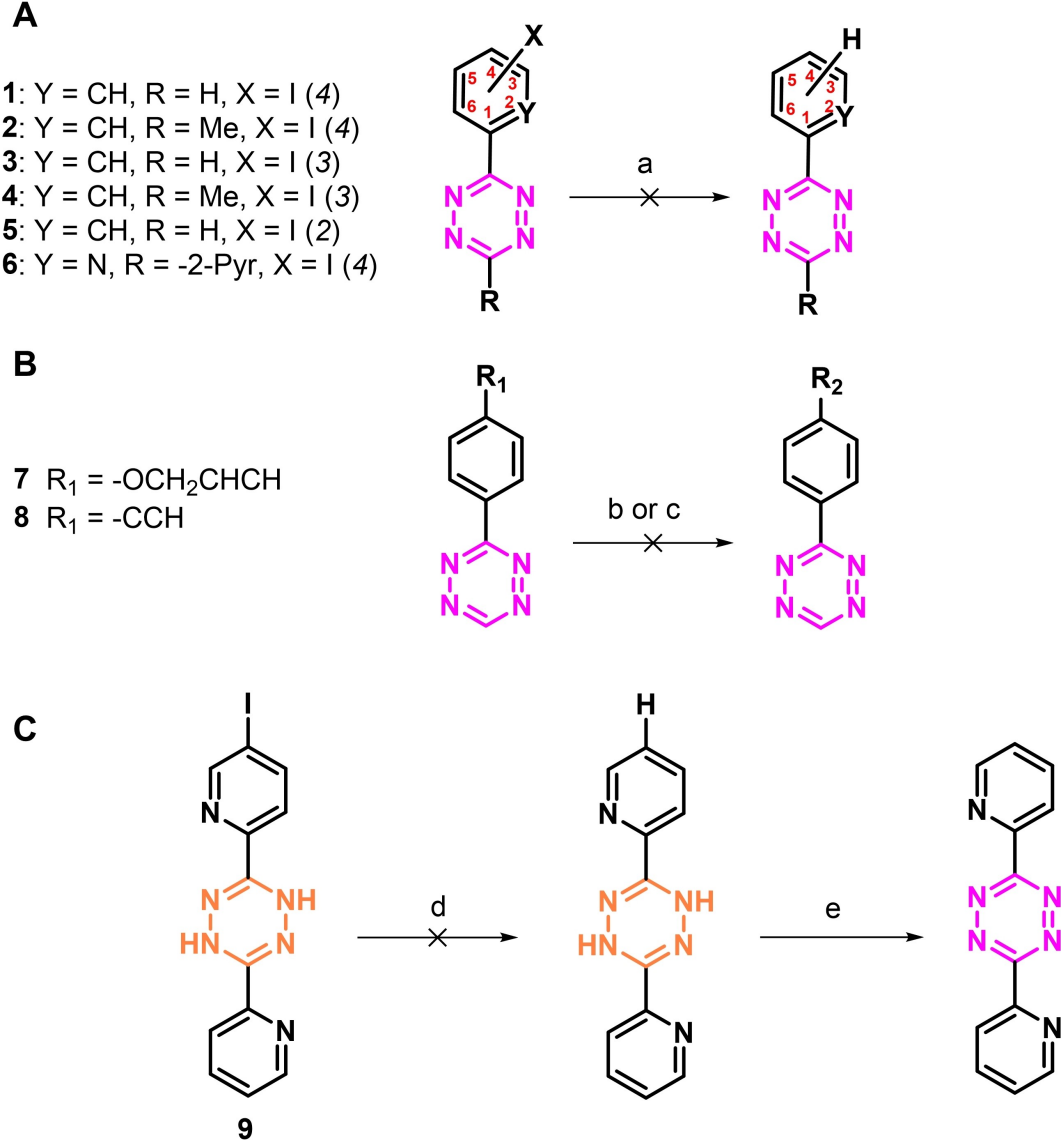
A) Reduction of different iodine‐substituted tetrazines with H_2_. Reagents and conditions: a) For further details please check Table S1 in the SI. B) Reduction of alkene group in 3‐(4‐(allyloxy)phenyl)‐1,2,4,5‐tetrazine **7** and alkyne group in 3‐(4‐ethynylphenyl)‐1,2,4,5‐tetrazine **8**. Reaction conditions: b) Pd/C, H_2_, R.T. to 100 °C, EtOH, 72 h; or Pd/C, H_2_, R.T. to 100 °C, TEA, EtOH, 72 h; or Pd/C, H_2_, HCO_2_NH_4_, R.T. to 100 °C, MeOH/THF, 72 h; c) Lindlars catalyst, quinoline, H_2_ (g), R.T. 72 h. C) Alternative reduction of bispyridyl tetrazine. Reagents and conditions: d) Pd/C, H_2_, R.T., EtOH, 72 h; 0 % or Pd/C, H_2_, R.T. to 100 °C, TEA, EtOH, 72 h; or Pd/C, H_2_, HCO_2_NH_4_, R.T. to 100 °C, MeOH/THF, 72 h; e) NaNO_2_, AcOH, H_2_O, rt, 10 min.

The feasibility to use tritium gas to radiolabel Tzs was evaluated using H_2_ (g) before attempting radioactive procedures. Reaction conditions were carefully screened with respect to catalyst, temperature, hydrogen source, solvents, and time. Various Tz precursors (e. g., methyl‐ Tzs, H‐Tzs or bispyridyl Tzs) substituted with an iodine atom at different positions were additional investigated. None of the applied procedures resulted in the desired product (Scheme 1A, SI Table S1). In a next step, alkene and alkyne bearing Tzs were unsuccessfully attempted to be reduced (Scheme 1B). Our results suggest that the tetrazine moiety is inhibiting the applied Pd‐catalyst. As Tzs are widely employed moieties to coordinate metals, the observed behavior is likely.^[7]^ To confirm this hypothesis the stable dihydro tetrazine derivative **9** was synthesized. However, again no reduction was observed suggesting that the Tz core even in the dihydro form can inhibit Pd‐catalyzed reduction.

### 2.2 Reduction with sodium borotritide (NaBT_4_)

**Scheme 2:**
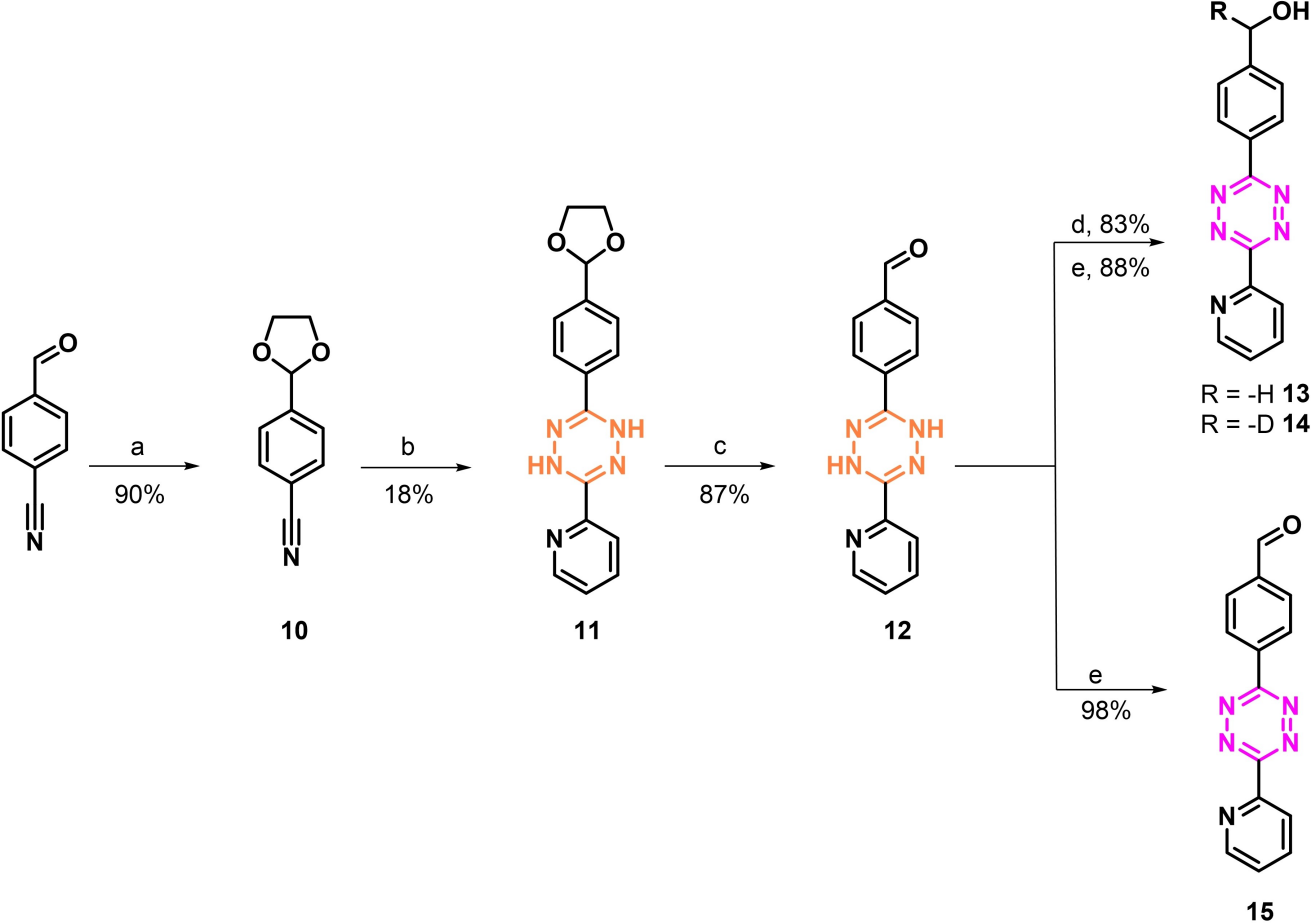
Reagents and conditions. a) Ethylene glycol, PTSA, toluene, reflux, 18 h; b) 2‐pyridinecarbonitrile, NH_2_NH_2_ ⋅ H_2_O, S_8_, EtOH, 90 °C, 12 h; c) H_2_O, DMSO, MW 110 °C, 1 h; d) NaBH_4_ or NaBD_4_, MeOH/CH_2_Cl_2_, rt, 5 min; e) NaNO_2_, AcOH, H_2_O, rt, 10 min.

Since all attempts failed to synthesize a ^3^H‐labeled tetrazine using Pd‐chemistry, we explored if reduction of an aldehyde with NaBT_4_ could be employed. We investigate this strategy first non‐radioactively using NaBH_4_. As the Tz core is known to be reduced to its corresponding dihydro analogues with NaBH_4_, we decided to start the reaction from the dihydro‐Tz, sequentially reduced the aldehyde group and oxidized then selectively the dihydro‐Tz to the Tz core.^[8]^ We selected a phenyl‐pyridyl Tz analogue in order to avoid autooxidation that is typically observed for diphenyl Tzs. Bispyridyl Tzs are known to be more reactive but less stable than phenyl‐pyridyl Tz and as such, we believed that phenyl‐pyridyl Tz display the best choice to develop a ^3^H‐labeled Tz derivative.^[9]^ It is known that the limited stability of tetrazines in biological media strongly correlates with the electron‐withdrawing effect of the substituents and for this reason bispyridyl Tzs undergo faster decomposition compared to phenyl‐pyridyl Tz.^[9]^ The synthesis of the precursor and the reference compounds are shown in Scheme 2. Briefly, 4‐formylbenzonitrile was converted to the corresponding acetal **10** under acidic catalysis in quantitative yields. The latter was reacted with an excess of 2‐carbonitrile and hydrazine hydrate to afford the dihydrotetrazine **11**.^[10]^ The dioxolane moiety was then hydrolyzed to the corresponding formyl compound **12** under microwave irradiation. Reduction with NaBH_4_ or NaBD_4_ followed by oxidation with NaNO_2_ afforded respectively Tz **13** and **14**. Differently direct oxidation of compound **12** gave aldehyde **15**.

**Figure 2:**
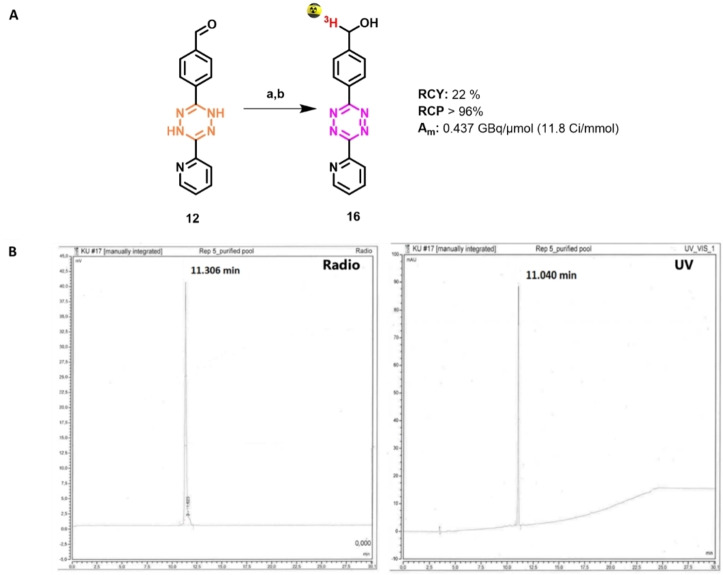
A) Tritiation of **12**: a) NaBT_4_, MeOH, CH_2_Cl_2,_ rt, 5 min. b) NaNO_2_, AcOH, H_2_O, rt, 10 min. B) HPLC chromatograms of the final product [^3^H]**16**.

Encouraged by these results, the tritiation of dihydro‐Tz **12** to Tz **16** was performed using the same reduction‐oxidation sequence (Figure 2). The product ^3^H‐Tz **16** was obtained with a radiochemical yield (RCY) of 22 %, a radiochemical purity (RCP) of 96 % and a molar activity (A_m_) of 0.437 GBq/μmol (11.8 Ci/mmol).^[11]^

### 2.3 Pretargeted autoradiography

**Figure 3:**
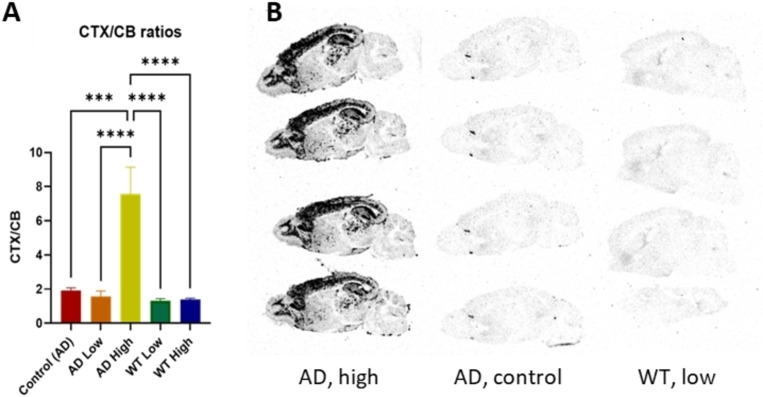
Results of the pretargeted autoradiography. A) CTX/CB ratio of [^3^H]**16** binding. Control=AD Brain, 0 μg/mL mAb‐TCO. AD low=AD brain, 0.006 μg/mL mAb‐TCO. AD High=AD brain, 0.06 μg/mL mAb‐TCO. WT low=WT brain, 0.006 μg/mL mAb‐TCO. WT High=WT brain, 0.06 μg/mL mAb‐TCO. Standard deviation calculated ANOVA. B) Autoradiographic image, showing the image contrast between AD High, AD control and WT low.

To determine the possibility of [^3^H]**16** to be used as a synthon, we performed pretargeted imaging. TCO‐mAb 3D6 (anti‐Abeta) was applied to brain of an amyloid‐beta (Aβ) pathology mouse model (Tg‐ArcSwe). After incubation of the Tg‐ArcSwe mice brain slices with TCO‐modified 3D6 overnight, excess antibody was washed away followed by incubation of the [^3^H]**16**. Significant amount of cortical (CTX) binding was observed in comparison to the cerebellum (CB), which is in agreement with the Aβ deposition pattern (Figure 3). Wild type and control animals showed a 4‐fold lower CTX/CB ratio.

## 3 Conclusion

In conclusion, a tritiation strategy was successfully developed for the labeling of Tz **16** using sodium borotritide. The compound was obtained in satisfactory RCY of 22 % and high RCP of >96 %. [^3^H]**16** was thereafter used for pretargeted autoradiography. Preincubation of TCO‐3D6 followed by application of [^3^H]**16** confirmed the ability of the [^3^H]**16** to be used for evaluation experiments. This study might pave the road to develop other tritiated Tzs which could even be used for *ex vivo* applications.

## Experimental Section

### Chemistry

#### General

All reagents and solvents were dried prior to use according to standard methods. Commercial reagents were used without further purification. Analytical TLC was performed using silica gel 60 F254 (Merck) with detection by UV absorption and/or by charring following immersion in a 7 % ethanolic solution of sulfuric acid or KMnO_4_‐solution (1.5 g of KMnO_4_, 10 g K_2_CO_3_, and 1.25 mL 10 % NaOH in 200 mL water). Purification of compounds was carried out by column chromatography on silica gel (40–60 μm, 60 Å) or employing a CombiFlash NextGen 300+ (Teledyne ISCO). ^1^H and ^13^C NMR spectra were recorded on Brucker (400 and 600 MHz instruments), using chloroform‐*d*, methanol‐*d*_4_ or DMSO‐*d*_6_ as deuterated solvent and with the residual solvent as the internal reference. For all NMR experiments the deuterated solvent signal was used as the internal lock. Chemical shifts are reported in δ parts per million (ppm). Coupling constants (*J* values) are given in Hertz (Hz). Multiplicities of ^1^H NMR signals are reported as follows: s, singlet; d, doublet; dd, doublet of doublets; ddd, doublet of doublets of doublets; dt, doublet of triplets; t, triplet; q, quartet; m, multiplet; br, broad signal. NMR spectra of all compounds are reprocessed in MestReNova software (version 12.0.22023) from original FID's files. Mass spectra analysis was performed using MS‐Acquity‐A: Waters Acquity UPLC with QDa‐detector. Purification by preparative HPLC was performed on Agilent 1260 infinity system, column SymmetryPrep‐C18, 17 mL/min H_2_O‐MeCN gradient 50–100 % 15 min with 0.1 % trifluoroacetic acid. All final compounds were >95 % pure as determined by analytical HPLC. Analytical HPLC method: (Thermo Fisher® UltiMate 3000) with a C‐18 column (Luna® 5u C18(2) 100 Å, 150×4.6 mm), eluents: A: H2O with 0.1 % TFA, B: MeCN with 0.1 % TFA. Gradient from 100 % A ‐>100 % B over 15minutes, back to 100 % A over 4 minutes, flow rate 1.5 mL/min. Detection by UV‐absorption at λ=254 nm on a UVD 170 U detector.

The modified antibody used in this study was kindly provided by Tagworks Pharmaceuticals. NaBT_4_ (specific activity: 2.96 TBq/mmol, 3.7 GBq, 1.25 μmol) was obtained from RC TRITEC AG (Teufen, Switzerland), stored as a solid in a sealed ampoule under argon gas at 750 mbar at room temperature. HPLC purification was carried out on a semi‐preparative C18 Luna Column (5 μm, 100 Å, 150×10 mm) and monitored with a 254 nm UV detector and a Raytest Gabi NaI detector. The solvent systems used were water (0.1 % TFA, solvent A) and acetonitrile (0.1 % TFA, solvent B) with a flow rate of 3 mL min^−1^. The RCY was determined by comparison of the starting activity which was present in the reaction mixture vial and the radioactivity present in the purified and formulated product. Radioactivity was measured by liquid scintillation counting. Molar activity was quantified by standard curve.

#### Previously described compounds

Tzs **1**–**6**, **8** and **9** were synthesized as previously described by Garcia *et al*. and Steen *et al*.[^4b^, ^12^]

#### Synthesis of 3‐(4‐(allyloxy)phenyl)‐1,2,4,5‐tetrazine (7)

The title compound was obtained from 4‐(allyloxy)benzonitrile (637 mg, 4.00 mmol) following the procedure from Garcia *et al*.[^4b^, ^12^] The tetrazine was purified via flash chromatography using *n*‐heptane: EtOAc (ethyl acetate) (95/5) as eluent, yielded as a pink solid (50 mg, 4 %). Rf=0.41 (*n*‐heptane: EtOAc: 9/1). ^1^H NMR (400 MHz, CDCl_3_) δ 10.12 (s, 1H), 8.64–8.43 (m, 2H), 7.16–7.00 (m, 2H), 6.17–5.91 (m, 1H), 5.55–5.39 (m, 1H), 5.39–5.27 (m, 1H), 4.66 (s, 2H).

#### 4‐(6‐(Pyridin‐2‐yl)‐1,4‐dihydro‐1,2,4,5‐tetrazin‐3‐yl)benzaldehyde (12)

**4‐(1,3‐dioxolan‐2‐yl)benzonitrile (10)**: The compound was synthesized accordingly to the previously published procedure Boyer *et al*.^[13]^ Ethylene glycol (3.07 mL, 54.90 mmol) and *p*‐toluene sulfonic acid (0.46 g, 2.44 mmol) were added into a solution of 4‐cyanobenzaldehyde (4 g, 30.50 mmol) in 150 mL of toluene. The reaction mixture was refluxed for 12 h and a Dean‐Stark trap was used to remove generated water during the reaction. After cooled to room temperature, 40 mL of a 5 % NaHCO_3_ aqueous solution was added. The organic layer was extracted with CH_2_Cl_2_, washed with water three times, and dried over Na_2_SO_4_. The solvent was removed under reduced pressure to yield 5.2 g (97 %) of 4‐(1,3‐dioxolan‐2‐yl)benzonitrile **10** as a white crystalline solid. Spectral data were identical to what was previously reported. Rf=0.35 (n‐heptane/EtOAc 70/30); ^1^H NMR (400 MHz, CDCl_3_) δ 7.67 (d, *J*=8.4 Hz, 2H), 7.59 (d, *J*=8.2 Hz, 2H), 5.84 (s, 1H), 4.86–2.69 (m, 4H).

**3‐(4‐(1,3‐Dioxolan‐2‐yl)phenyl)‐6‐(pyridin‐2‐yl)‐1,4‐dihydro‐1,2,4,5‐tetrazine (11)**: The compound was synthesized accordingly to the previously published procedure.^[10]^ 4‐(1,3‐Dioxolan‐2‐yl)benzonitrile (1.12 g, 6.39 mmol) 2‐cyanopyridine (5.32 g, 51.14 mmol) were dissolved in 100 mL of ethanol with hydrazine hydrate (6.67 mL, 95.89 mmol) and warmed to 60 °C. After 15 minutes, sulfur (410 mg, 1.59 mmol) was added, and the solution refluxed for 12 h. The mixture was cooled to room temperature and the ethanol was removed under reduced pressure. Purification by flash chromatography (CH_2_Cl_2_/MeOH 95/5) afforded 0.35 g (18 %) of the desired product as a yellow solid. Rf=0.48 (n‐Heptane/EtOAc 50/50); ^1^H NMR (400 MHz, CDCl_3_) δ 8.78–8.46 (m, 2H), 8.04 (dt, *J*=8.0, 1.1 Hz, 1H), 7.78 (td, *J*=7.8, 1.7 Hz, 1H), 7.57 (d, *J*=8.3 Hz, 2H), 7.38 (ddd, *J*=7.6, 4.9, 1.2 Hz, 2H), 7.18 (s, 1H), 5.87 (s, 1H), 4.28–4.00 (m, 2H); ^13^C NMR (101 MHz, CDCl_3_) δ 148.43, 147.35, 147.25, 147.13, 140.44, 136.76, 130.94, 126.94, 125.95, 124.99, 121.22, 103.06, 65.35; HPLC‐MS [M+H]^+^ *m*/*z*: calcd for [C_16_H_16_N_5_O_2_]^+^, 310.1; found, 310.1.

**4‐(6‐(Pyridin‐2‐yl)‐1,4‐dihydro‐1,2,4,5‐tetrazin‐3‐yl)benzaldehyde (12)**: 3‐(4‐(1,3‐Dioxolan‐2‐yl)phenyl)‐6‐(pyridin‐2‐yl)‐1,4‐dihydro‐1,2,4,5‐tetrazine (0.12 g, 0.39 mmol) was added to a microwave vial equipped with a stir bar which was then sealed and purged with N_2_. Water (6 mL) and DMSO (1 mL) were added via a syringe and the reaction allowed to stir at 110 °C in a microwave for 1 hour. The reaction was allowed to cool to room temperature and unsealed. The suspension was extracted with CH_2_Cl_2_ washed with brine, dried over MgSO_4_, filtered and concentrated under reduced pressure. Purification by flash chromatography (n‐Heptane/EtOAc 60/40) afforded 0.09 g (87 %) of the desired compound as a yellow solid. Rf=0.38 (n‐Heptane/EtOAc 50/50); ^1^H NMR (400 MHz, DMSO) δ 10.06 (s, 1H), 9.44 (s, 1H), 8.91 (s, 1H), 8.65 (dd, *J*=4.8, 1.5 Hz, 1H), 8.04 (d, *J*=8.2 Hz, 2H), 8.00–7.89 (m, 4H), 7.54 (ddd, *J*=6.5, 5.0, 1.5 Hz, 1H); ^13^C NMR (101 MHz, DMSO) δ 193.10, 149.08, 147.62, 147.36, 146.78, 137.88, 137.50, 135.68, 130.06, 127.15, 125.82, 121.47; HPLC‐MS [M+H]^+^ *m*/*z*: calcd for [C_14_H_12_N_5_O]^+^, 266.1; found, 266.1.

#### Synthesis of (4‐(6‐(pyridin‐2‐yl)‐1,2,4,5‐tetrazin‐3‐yl)phenyl)methanol (13)

To a solution of 4‐(6‐(pyridin‐2‐yl)‐1,4‐dihydro‐1,2,4,5‐tetrazin‐3‐yl)benzaldehyde (0.03 g, 0.11 mmol) in MeOH/CH_2_Cl_2_ (6 mL, 50/50 v/v) was added NaBH_4_ (0.005 g, 0.13 mmol). The reaction was stirred at room temperature for 10 minutes. A solution of NaNO_2_ (0.08 g, 1.13 mmol) in water (5 mL) was added to the mixture. Glacial acetic acid (3 mL) was added, and the reaction turned bright red. The mixture was stirred for 30 minutes and subsequently extracted with CH_2_Cl_2_ washed with brine, dried over MgSO_4_, filtered and concentrated under reduced pressure. Purification by flash chromatography (CH_2_Cl_2_/MeOH 95/5) afforded 0.025 g (83 %) of the desired compound as a red solid. Rf=0.33 (CH_2_Cl_2_/MeOH 98/2); ^1^H NMR (600 MHz, MeOD) δ 8.76 (d, *J*=4.9 Hz, 1H), 8.64 (dd, *J*=8.0, 1.3 Hz, 1H), 8.55 (d, *J*=8.3 Hz, 1H), 8.05 (td, *J*=7.8, 1.7 Hz, 1H), 7.61 (ddd, *J*=7.5, 4.8, 1.2 Hz, 1H), 7.56 (d, *J*=8.0 Hz, 1H), 4.66 (s, 2H); ^13^C NMR (151 MHz, MeOD) δ 164.46, 163.04, 150.08, 149.99, 147.15, 138.05, 130.61, 127.97, 127.13, 126.59, 123.82, 63.22; HPLC‐MS [M+H]^+^ *m*/*z*: calcd for [C_14_H_12_N_5_O]^+^, 266.1; found, 266.1.

#### Synthesis of (4‐(6‐(pyridin‐2‐yl)‐1,2,4,5‐tetrazin‐3‐yl)phenyl)methan‐d–ol (14)

To a solution of 4‐(6‐(Pyridin‐2‐yl)‐1,4‐dihydro‐1,2,4,5‐tetrazin‐3‐yl)benzaldehyde (0.017 g, 0.06 mmol) in MeOH/CH_2_Cl_2_ (6 mL, 50/50) was added NaBD_4_ (0.003 g, 0.07 mmol). The reaction was stirred at room temperature for 10 minutes. A solution of NaNO_2_ (0.044 g, 0.64 mmol) in water (5 mL) was added to the mixture. Glacial acetic acid (3 mL) was added, and the reaction turned bright red. The mixture was stirred for 30 minutes and subsequently extracted with CH_2_Cl_2_ washed with brine, dried over MgSO_4_, filtered and concentrated under reduced pressure. Purification by flash chromatography (CH_2_Cl_2_/MeOH 95/5) afforded 0.015 g (88 %) of the desired compound as a red solid. Rf=0.35 (CH_2_Cl_2_/MeOH 98/2); ^1^H NMR (600 MHz, CDCl_3_) δ 9.07–8.92 (m, 1H), 8.74–8.64 (m, 3H), 8.00 (td, *J*=7.7, 1.8 Hz, 1H), 7.61 (d, *J*=8.2 Hz, 2H), 7.57 (ddd, *J*=7.6, 4.7, 1.2 Hz, 1H), 4.84 (t, *J*=1.9 Hz, 1H); ^13^C NMR (151 MHz, CDCl_3_) δ 164.20, 163.38, 150.90, 150.31, 146.28, 137.47, 130.67, 128.61, 127.47, 126.34, 123.92, 64.45 (t, *J*=21.9 Hz); HPLC‐MS [M+H]^+^ *m*/*z*: calcd for [C_14_H_11_DN_5_O]^+^, 267.1; found, 267.1.

#### Synthesis of 4‐(6‐(pyridin‐2‐yl)‐1,2,4,5‐tetrazin‐3‐yl)benzaldehyde (15)

To a solution of 4‐(6‐(Pyridin‐2‐yl)‐1,4‐dihydro‐1,2,4,5‐tetrazin‐3‐yl)benzaldehyde (0.05 g, 0.18 mmol) in MeOH/CH_2_Cl_2_ (6 mL, 50/50) was added a solution of NaNO_2_ (0.13 g, 1.88 mmol) in water (5 mL). Glacial acetic acid (3 mL) was added, and the reaction turned bright red. The mixture was stirred for 30 minutes and subsequently extracted with CH_2_Cl_2_ washed with brine, dried over MgSO_4_, filtered and concentrated under reduced pressure. Purification by flash chromatography (CH_2_Cl_2_/MeOH 95/5) afforded 0.045 g (91 %) of the desired compound as a red solid. Rf=0.27 (CH_2_Cl_2_/MeOH 99/1); ^1^H NMR (400 MHz, CDCl_3_) δ 10.18 (s, 1H), 9.04–8.97 (m, 1H), 8.89 (d, *J*=8.3 Hz, 2H), 8.74 (dd, *J*=8.0, 1.1 Hz, 1H), 8.14 (d, *J*=8.4 Hz, 2H), 8.03 (td, *J*=7.8, 1.8 Hz, 1H), 7.60 (ddd, *J*=7.7, 4.8, 1.2 Hz, 1H); ^13^C NMR (101 MHz, CDCl_3_) δ 191.57, 163.84, 163.58, 151.11, 150.01, 139.18, 137.53, 136.73, 130.36, 128.95, 126.65, 124.28; HPLC‐MS [M+H]^+^ *m*/*z*: calcd for [C_14_H_10_N_5_O]^+^, 264.1; found, 264.1.

### Radiolabeling [^3^H]‐(4‐(6‐(pyridin‐2‐yl)‐1,2,4,5‐tetrazin‐3‐yl)phenyl)methan‐t–ol (16)

To 100 μL of a solution of the precursor **12** (3 mg/mL in DCM, 130 μL, 1.5 μmol, 1.2 eq.) was added 100 μL of NaBT_4_ (3.7 GBq, 1.25 μmol, 1 eq.) solution in MeOH was added. The reaction was stirred for 5 minutes at room temperature. The reaction was monitored with HPLC and after 5 minutes, a conversion was observed from precursor **12** (RT=12.9 min) to the reduced intermediate (RT=10.5 min). A saturated solution of NaNO_2_ was added in excess. The reaction was acidified with AcOH until the color changed to red (pH 2–3) to fully oxidize the compound to the Tz **16**. The DCM layer was extracted twice, and the crude was injected on analytical HPLC, showing a full conversion to product **16** (RT=11.3 min). The crude was purified with semi‐prep HPLC. The product was [^3^H]**16** with an RCY of 22 %, based on liquid scintillation counting, RCP of 96 %, based on radio‐HPLC and a molar activity of 11.8 Ci/mmol.

### Pretargeted autoradiography

All animal procedures were performed in accordance with the European Commission's Directive 2010/63/EU, as well as the ARRIVE guidelines, and were approved by the Danish Council of Animal Ethics (Journal no. 2017‐15‐0201‐01375). Autoradiography was performed using 20 μm cryosections containing sagittal sections of Tg‐ArcSwe mice brains. Frozen brains were sectioned on a cryostat (Leica CM1800, Leica Biosystems, Buffalo Grove, IL, United States) and mounted on Superfrost Plus™ adhesion microscope slides (Thermo Fischer Scientific, MS, United States). Sections were stored at −80 °C for the remaining period of the study. The sections were thawed at 4 °C before the study, and consequently pre‐incubated with PBS containing 1 % BSA (700 mL/slide) for 30 minutes. Buffer was removed from the slides and PBS containing 0.05 % TWEEN‐20 was added to the slides (700 μL/slide) and incubated for 5 minutes. After 5 minutes two concentrations of TCO‐mAb 3D6 solution was added to the slides, the high concentration being 0.06 μg/mL mAb‐TCO and the low concentration=0.006 μg/mL mAb‐TCO, this was incubated for 24 h. After 24 h excess TCO‐mAb was removed by washing in cold PBS (3×15 min) and cold distilled water (1×30 s). Consequently, 20 nM of radiolabeled [^3^H]**16** in PBS containing 0.05 % TWEEN‐20 was added to all slides (700 μL/slide) and incubated for 1 h at room temperature. Non‐specific binding was determined with two control slides of WT brain slices and one slide containing Tg‐ArcSwe mice brain with no mAb‐TCO incubated but PBS. Excess radioactive solution was washed with cold distilled water (1×30 s). The slides were subjected to a gentle airflow and fixated overnight in a paraformaldehyde vapor chamber in cold storage (4 °C). After 24 h the slides were moved to an exicator for 1 h to remove any excess moisture and then arranged for exposure to a tritium sensitive imaging plate (BAS‐IP TR2040, Science Imaging Scandinavia AB, Nacka, Sweden), together with tritium standards (RPA510, Amersham Bioscience, GE Healthcare, Chicago, IL, USA. After six days of exposure the plate was read out by a Fujifilm BAS 1000 scanner (RPA510, Amersham Bioscience, GE Healthcare, Chicago, IL, USA). Analysis and quantification of the autoradiography were performed using OptiQuant and Excel respectively. The four‐parameter general curve fit (David Rodbard, NIH) of decay corrected tritium standards was used to convert mean pixel density (grayscale) to nCi/mg tissue equivalent (TE).

## Author Contributions

Conceptualization (lead): M. M. H., U. B. Chemistry: U. B. Labeling: N. B. and A. M. D. Autoradiography: N. B., V. S. and S. L. B. Original draft preparation, review and editing: N. B., U. B. Review and editing: N. B., U. B., M. M. H. All authors read and approved the final manuscript.

## Conflict of interest

The authors declare no conflict of interest.

4

## Supporting information

As a service to our authors and readers, this journal provides supporting information supplied by the authors. Such materials are peer reviewed and may be re‐organized for online delivery, but are not copy‐edited or typeset. Technical support issues arising from supporting information (other than missing files) should be addressed to the authors.

Supporting Information

## Data Availability

Data will be made available on request.
