# Supplementary material for: Development of the First Tritiated Tetrazine: Facilitating Tritiation of Proteins
Source: Chembiochem. 2022 Nov 4;23(23):e202200539. doi: 10.1002/cbic.202200539 (PMC10100488; doi:10.1002/cbic.202200539)
Supplement: Supplementary file 1 — Supporting Information [file CBIC-23-0-s001.pdf]

ChemBioChem

Supporting Information

Development of the First Tritiated Tetrazine: Facilitating Tritiation of Proteins

Natasha Shalina Radjani Bidesi[+], Umberto Maria Battisti[+], Sara Lopes van de Broek, Vladimir Shalgunov, Anne-Mette Dall, Jesper Bøggild Kristensen, Dag Sehlin, Stina Syvänen, Gitte Moos Knudsen, and Matthias Manfred Herth*

Table of Contents

1. Radiolabeling S2

2. HPLC Traces S6

3. Pretargeted Autoradiography S9

4. NMR Spectra S12

2. Radiolabeling

Table S1. Reagents and conditions for Pd-catalyzed hydrodehalogenations of Tz **1-6**.

1: Y = CH, R = H, X = I (*4*)
2: Y = CH, R = Me, X = I (*4*)
3: Y = CH, R = H, X = I (*3*)
4: Y = CH, R = Me, X = I (*3*)
5: Y = CH, R = H, X = I (*2*)
6: Y = N, R = -2-Pyr, X = I (*4*)

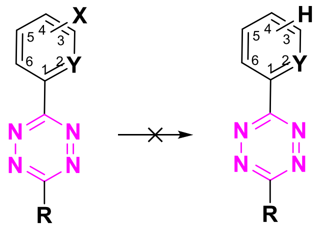

1-6

Entry	Reagent	Catalyst	H-source	Base/Acid	Solvent	Temperature	Time	Conversion	Ref
1	**1-6**	Pd/C	H_2 (g)	-	EtOH	R.T. to 100 ^{o}C	1h-3d	-	[1]
2	**1-6**	Pd/C	H_2 (g)	TEA	EtOH	R.T. to 100 ^{o}C	1h-3d	-	[1a, 2]
3	**1-6**	Pd/C	H_2 (g)	TEA	MeOH	R.T. to 100 ^{o}C	1h-3d	-	[1a, 2]
4	**1-6**	Pd/C	HCO_2NH_4	-	MeOH/THF	R.T. to 100 ^{o}C	1h-3d	-	[3]
5	**1,2,6**	Pd/C	H_2 (g)	NH_4OH	MeOH	R.T. to 100 ^{o}C	1h-3d	-	[4]
6	**1,2,6**	Pd/C	H_2 (g)	Hunig's base	DMF	R.T. to 100 ^{o}C	1h-3d	-	[5]
7	**1,2,6**	Pd/C	H_2 (g)	-	THF	R.T. to 100 ^{o}C	1h-3d	-	-
8	**1-6**	PtO_2	H_2 (g)	-	EtOH	R.T. to 100 ^{o}C	1h-3d	-	[1a, 6]
9	**1-6**	$Pd(dba)_2$	H_2 (g)	-	MeOH	R.T. to 100 ^{o}C	1h-3d	-	-
10	**1-6**	$Pd(dba)_2$ SiMes.HCl, KOMe	H_2 (g)	KOMe	dioxane	100 ^{o}C	1h-3d	-	[7]
11	**1-6**	$Pd(PPh_3)_4$	H_2 (g)	HCO_2Na	MeOH	R.T. to 100 ^{o}C	1h-3d	-	[8]
12	**1,2,6**	$Pd(PPh_3)_4$	H_2 (g)	-	THF	R.T. to 100 ^{o}C	1h-3d	-	[8]
13	**1-6**	$PdCl_2$	TMDS	-	H_2O	R.T.	1h-3d	-	[9]

General reduction entry 1

To a solution of Tz **1-6** (10 mg) in ethanol (0.1 mL) was added Pd/C (10 wt.%, 1 mg) at argon atmosphere. Consequently, the reaction mixture was charged with hydrogen while the flask was connected to a vacuum line. The mixture was stirred at room temperature for 24 h and monitored frequently. After 24 hours the temperature was raised to 100 °C, and monitored for 48 h. For all 6 Tzs no reduction was observed, based on LC-MS, TLC and NMR after filtration of the Pd/C, evaporation of the solvent.

General reduction entry 2

To general reduction entry 1, 4 µL trimethylamine was added. For all 6 Tzs no reduction was observed, based on LC-MS, TLC and NMR after filtration of the Pd/C, evaporation of the solvent.

General reduction entry 3

To general reduction entry 1, instead of ethanol, methanol was used. Caution was taken that all glassware and stirring bars were vacuum oven dried before use, and glassware was purged 3 x with argon. For all 6 Tzs no reduction was observed, based on LC-MS, TLC and NMR after filtration of the Pd/C, evaporation of the solvent. Hence, the compounds

General reduction entry 4

To a solution of Tz **1-6** (10 mg) in ethanol (0.1 mL) was added 1 ml of a saturated solution of HCO_2NH_4 (40-50 mg of HCO_2NH_4 in MeOH/THF, 2/1). Subsequently, Pd/C (10 wt.%, 1 mg) was added to the flask at argon atmosphere. The mixture was stirred at room temperature for 24 h and monitored frequently. After 24 hours the temperature was raised to 150 °C, and monitored for 48 h. For all 6 Tzs no reduction was observed, based on LC-MS, TLC and NMR after filtration of the Pd/C and drying of the filtrate in vacuo.

General reduction entry 5

To general reduction entry 3, 40 µl of HCl in dioxane (8M) was added. For all 3 Tzs (**1**, **2**, **6**) no reduction was observed, based on LC-MS, TLC and NMR after filtration of the Pd/C, evaporation of the solvent.

General reduction entry 6

To general reduction entry 3, 4 μL saturated $NaHCO_3$ was added. THF was used as solvent instead of MeOH. For all 3 Tzs (**1**, **2**, **6**) no reduction was observed, based on LC-MS, TLC and NMR after filtration of the Pd/C, evaporation of the solvent.

General reduction entry 7

To general reduction entry 1, instead of ethanol, THF was used. For all 3 Tzs (**1**, **2, 6**) no reduction was observed, based on LC-MS, TLC and NMR after filtration of the Pd/C, evaporation of the solvent.

General reduction entry 8

To general reduction entry 1, instead of Pd/C, 10 wt% PtO_2 was used. For all 6 Tzs no reduction was observed, based on LC-MS, TLC and NMR after filtration of the PtO_2, evaporation of the solvent.

General reduction entry 9

To general reduction entry 1, instead of Pd/C, 10 wt% $Pd(dba)_2$ was used. For all 6 Tzs no reduction was observed, based on LC-MS, TLC and NMR after filtration of the PtO_2, evaporation of the solvent.

General reduction entry 10

To a solution of 10 mg (1 eq.) Tz **1-6** was added $Pd(dba)_2$ (0.02 eq.), SIMes.HCl (0.02 eq.), KOMe (2 eq.), 100 mL of dioxane. Reaction vial was closed and purged with argon. Consequently, the reaction mixture was heated until 100 ^oC. For all 6 Tzs no reduction was observed, based on LC-MS, TLC and NMR after filtration of the PtO2, evaporation of the solvent.

General reduction entry 11

To a solution of 10 mg (1 eq.) Tz **1-6** was added $Pd(PPh_3)_4$ (0.10 eq.), HCOONa (0.2 eq.), 100 mL of MeOH. Reaction vial was closed and purged with argon. Consequently, the reaction mixture was heated until 100 ^oC. For all 11 Tzs no reduction was observed, based on LC-MS and TLC.

General reduction entry 12

To a solution of 10 mg (1 eq.) Tz **1-6** was added $Pd(PPh_3)_4$ (0.10 eq.) in 100 mL of MeOH. Reaction vial was closed and purged with argon. Consequently, the reaction mixture was heated until 100 ^oC. Consequently, the reaction mixture was charged with hydrogen while the flask was connected to a vacuum line. The mixture was stirred at room temperature for 24 h and monitored frequently. After 24 hours the temperature was raised to 100 °C, and monitored for 48 h. For all 6 Tzs no reduction was observed, based on LC-MS and TLC.

General reduction entry 13

To a solution of Tz **1-6** (10 mg) in deionized water (0.1 mL) was added $PdCl_2$ (0.05 eq.). The reaction mixture was stirred for 10 min. To the reaction mixture was added TMDS (0.4 eq.), dropwise. The mixture was stirred at room temperature for 3 days and monitored frequently. For all 6 Tz's no reduction was observed, based on LC-MS and TLC.

3-(4-propoxyphenyl)-1,2,4,5-tetrazine

Tz **7** was subjected to general reduction entry 1, 2 and 4. No reduction was observed, based on LC-MS, TLC and NMR after filtration of the Pd/C, and evaporation of the solvent.

3-(4-vinylphenyl)-1,2,4,5-tetrazine

To **8** (50 mg, 0.27 mmol), dissolved in 2 mL MeOH was added 2.5 mg of Lindlar catalyst and 25 mg of quinoline. Hydrogen gas contained in a balloon was bubbled into the reaction mixture at room temperature for 3 days. Reaction was monitored by TLC and LC-MS, however after 5 days no conversion was observed yet.

3,6-di(pyridin-2-yl)-1,2-dihydro-1,2,4,5-tetrazine

Tz **9** was subjected to general reduction entry 1,2 and 4. No reduction was observed, based on LC-MS, TLC and NMR after filtration of the Pd/C, and evaporation of the solvent. Hence the product **14** or **12** were never obtained.

*SI-2-1 HPLC Chromatograms of Precursor and References **12-15** plus dihydro-intermediate*

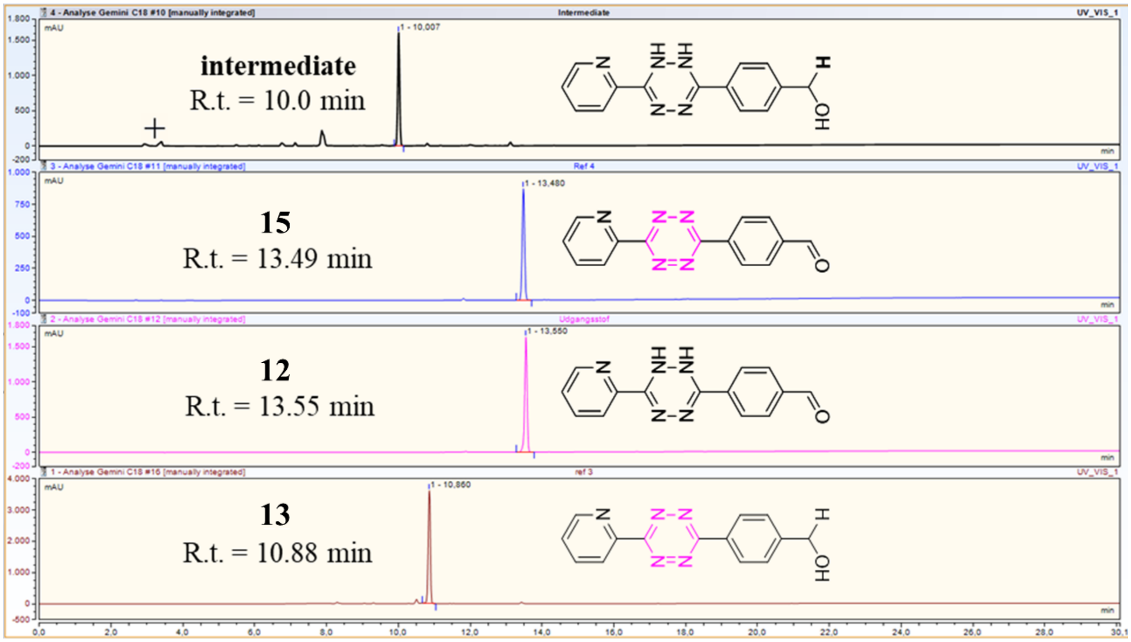

*SI-2-2 Crude ^{3}H-Tz **16** Radio-HPLC Chromatogram*

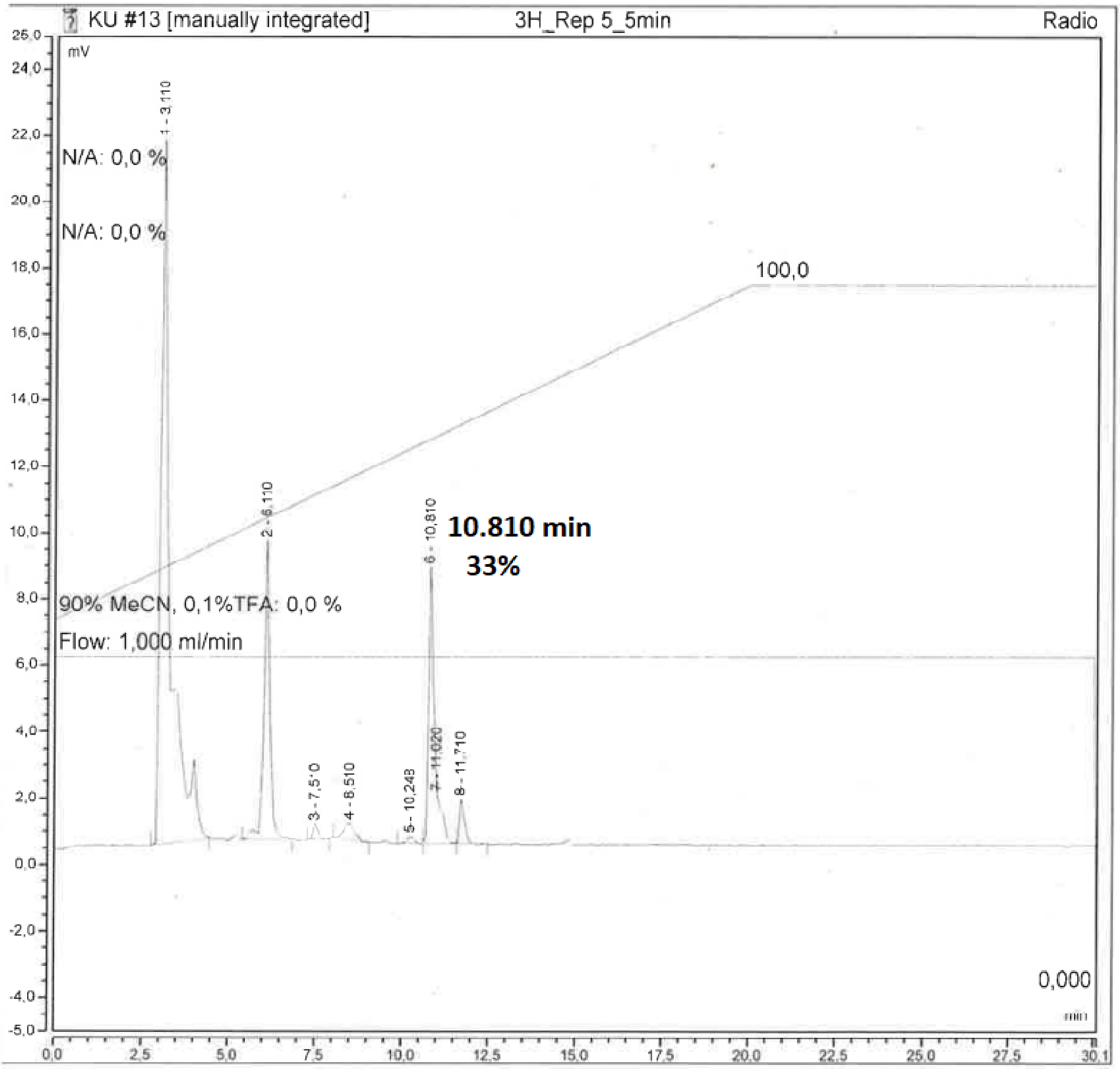

SI-2-3 Crude ^{3}H-Tz ***16*** *UV-HPLC Chromatogram*

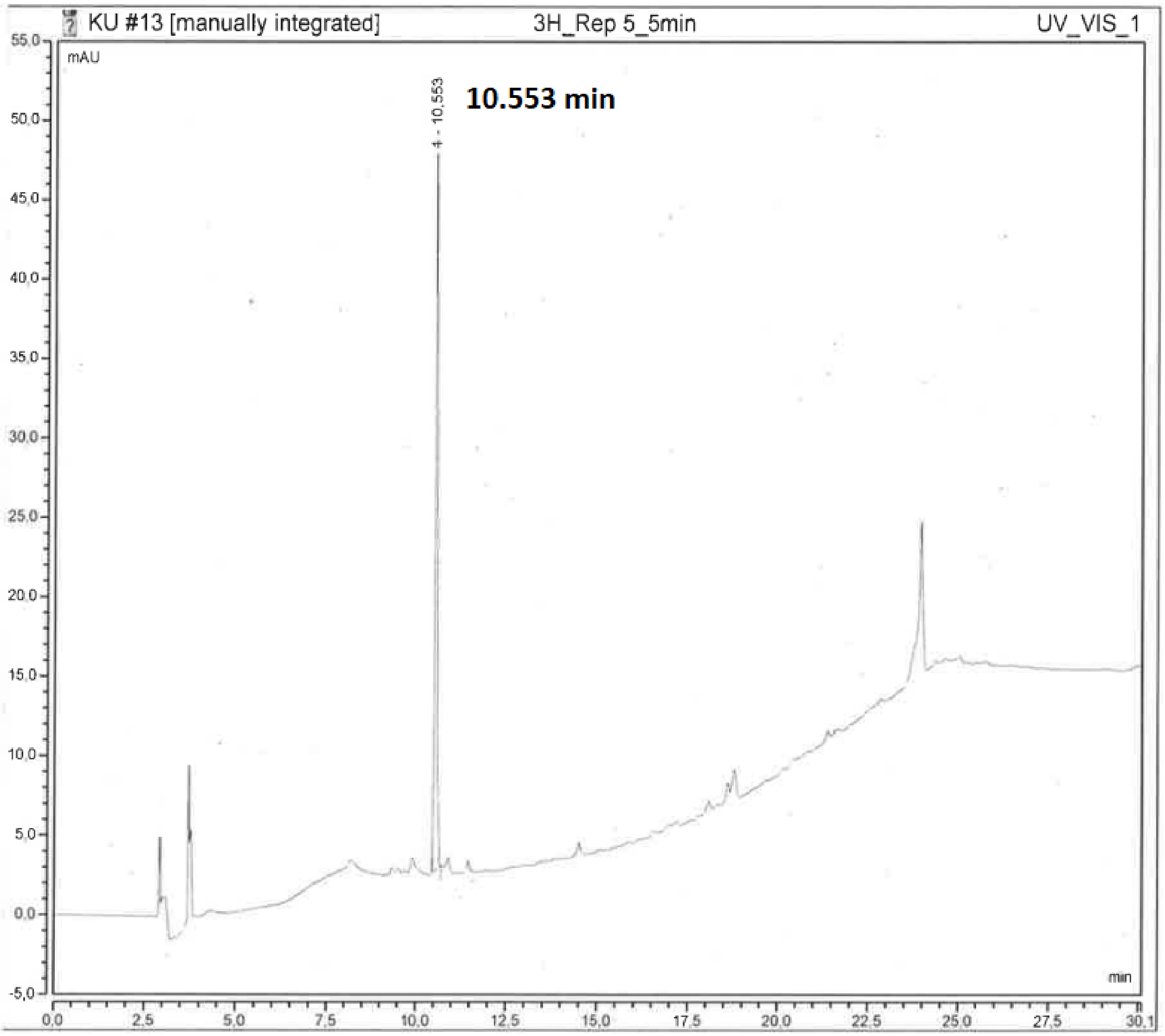

3. Pretargeted Autoradiography

SI-3-1 Raw data (fmol/mg) autoradiography. Calculated in GraphPad Prism, standard deviation calculated by ANOVA.

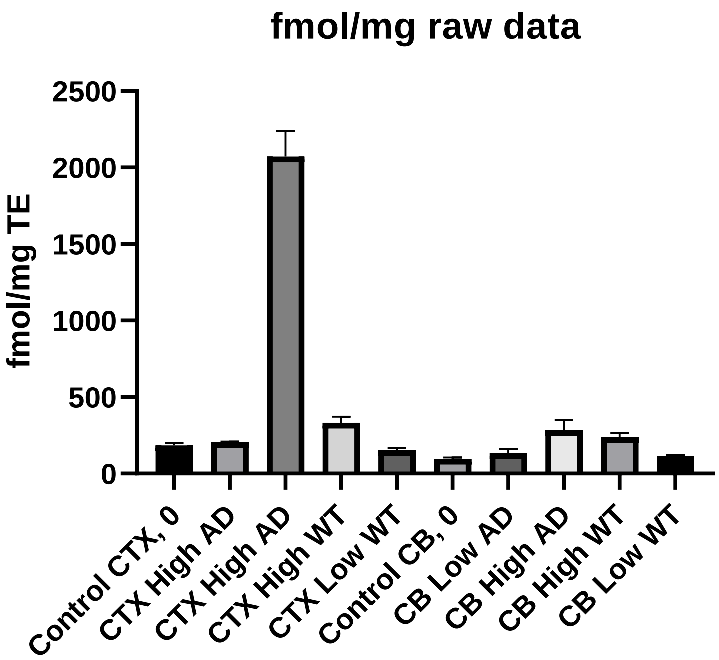

SI-3-2 Autoradiographic image as obtained in Image J

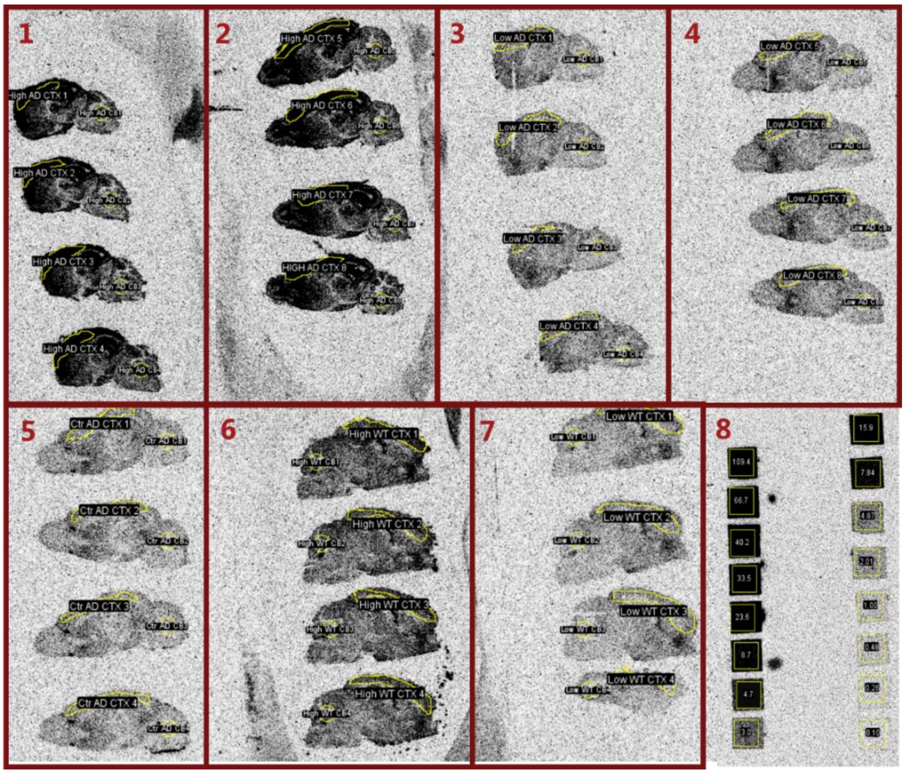

1: AD High 2: AD High 3: AD low 4: AD Low 5: AD Control 6: WT High 7: WT Low

SI-3-3 Raw Data of pretargeting autoradiography ^{3}***H-Tz 16*** *as calculated by Image J.*

**High AD CTX 1**		***0.040***	***24,635***
**HIGH AD CTX 2**		**0.039**	**25,641**
**HIGH AD CTX 3**		**0.053**	**24,940**
**HIGH AD CTX 4**		**0.056**	**21,291**
**HIGH AD CTX 5**		**0.059**	**24,460**
**HIGH AD CTX 6**		**0.071**	**24,488**
**HIGH AD CTX 7**		**0.058**	**28,652**
**HIGH AD CTX 8**		**0.054**	**24,466**
**LOW AD CTX 1**		**0.058**	**2,363**
**LOW AD CTX 2**		**0.059**	**2,469**
**LOW AD CTX 3**		**0.030**	**2,526**
**LOW AD CTX 4**		**0.059**	**2,544**
**LOW AD CTX 5**		**0.065**	**2,484**
**LOW AD CTX 6**		**0.069**	**2,404**
**LOW AD CTX 7**		**0.055**	**2,441**
**LOW AD CTX 8**		**0.071**	**2,406**
**CTR AD CTX 1**		**0.047**	**2,292**
**CTR AD CTX 2**		**0.050**	**2,389**
**CTR AD CTX 3**		**0.035**	**2,237**
**CTR AD CTX 4**		**0.064**	**1,962**
**HIGH WT CTX 1**		**0.071**	**4,688**
**HIGH WT CTX 2**		**0.060**	**3,874**
**HIGH WT CTX 3**		**0.066**	**3,645**
**HIGH WT CTX 4**		**0.076**	**3,745**
**LOW WT CTX 1**		**0.097**	**2,049**
**LOW WT CTW 2**		**0.089**	**1,836**
**LOW WT CTX 3**		**0.106**	**1,836**
**LOW WT CTX 4**		**0.041**	**1,584**
**High AD CB 1**		**0.018**	**3,338**
**HIGH AD CB 2**		**0.018**	**3,973**
**HIGH AD CB 3**		**0.018**	**2,992**
**HIGH AD CB 4**		**0.018**	**4,935**
**HIGH AD CB 5**		**0.018**	**3,132**
**HIGH AD CB 6**		**0.018**	**2,630**
**HIGH AD CB 7**		**0.017**	**3,584**

**HIGH AD CB 8**	**0.018**	**2,753**
**LOW AD CB 1**	**0.018**	**1,777**
**LOW AD CB 2**	**0.018**	**1,917**
**LOW AD CB 3**	**0.018**	**1,918**
**LOW AD CB 4**	**0.018**	**1,147**
**LOW AD CB 5**	**0.018**	**1,378**
**LOW AD CB 6**	**0.018**	**1,416**
**LOW AD CB 7**	**0.018**	**1,709**
**LOW AD CB 8**	**0.018**	**1,700**
**CTR AD CB 1**	**0.018**	**1,266**
**CTR AD CB 2**	**0.018**	**1,103**
**CTR AD CB 3**	**0.018**	**1,226**
**CTR AD CB 4**	**0.018**	**1,090**
**HIGH WT CB 1**	**0.018**	**3,304**
**HIGH WT CB 2**	**0.018**	**2,914**
**HIGH WT CB 3**	**0.018**	**2,676**
**HIGH WT CB 4**	**0.018**	**2,533**
**LOW WT CB 1**	**0.018**	**1,431**
**LOW WT CB 2**	**0.018**	**1,312**
**LOW WT CB 3**	**0.018**	**1,475**
**LOW WT CB 4**	**0.018**	**1,374**

1. NMR Spectra

3-(4-(allyloxy)phenyl)-1,2,4,5-tetrazine (7)

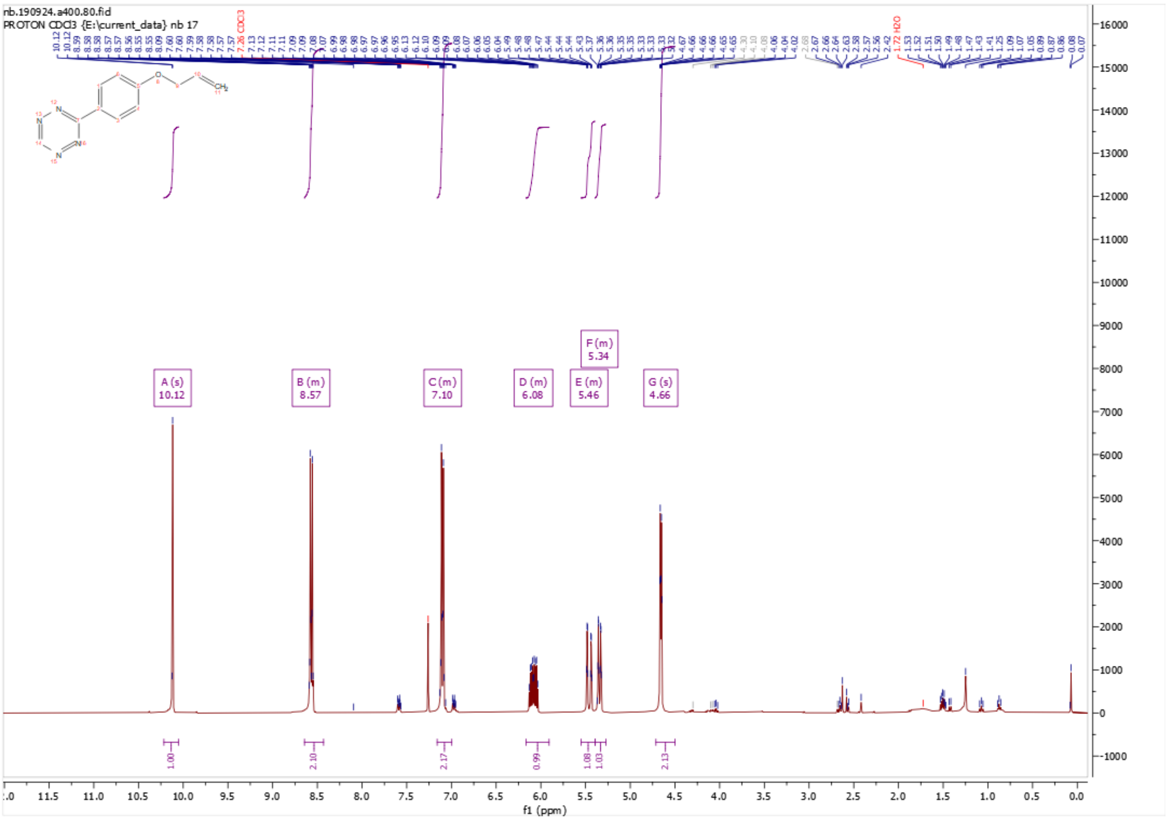

4-(1,3-dioxolan-2-yl)benzonitrile (10)

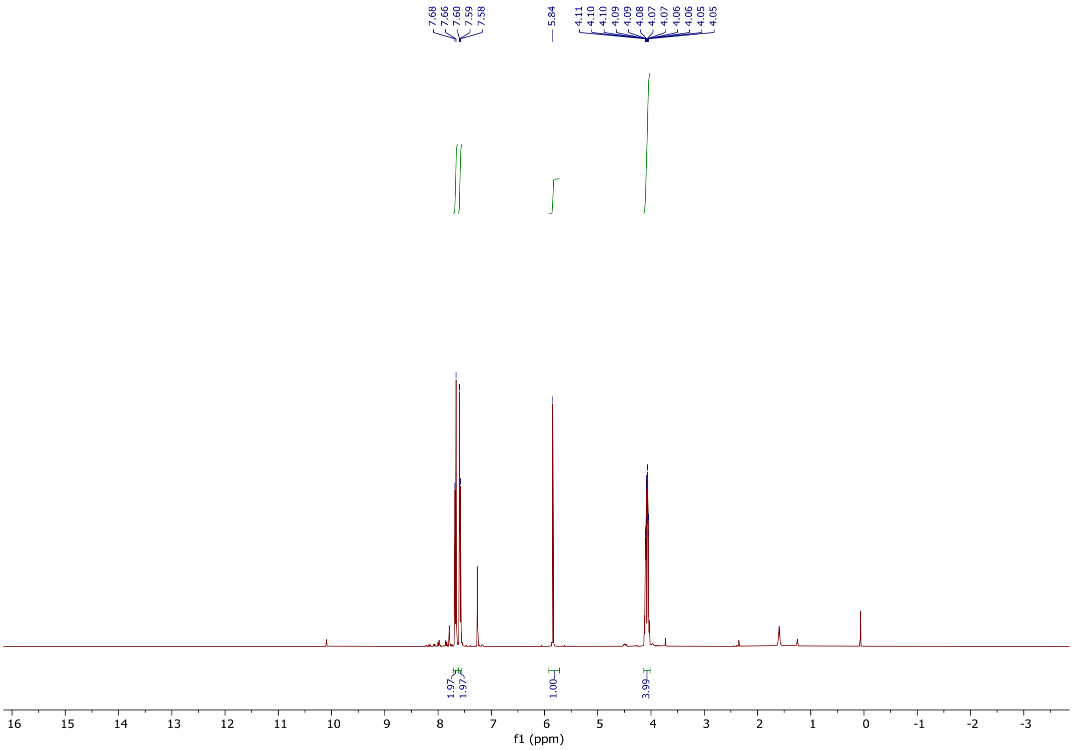

3-(4-(1,3-dioxolan-2-yl)phenyl)-6-(pyridin-2-yl)-1,4-dihydro-1,2,4,5-tetrazine (11)

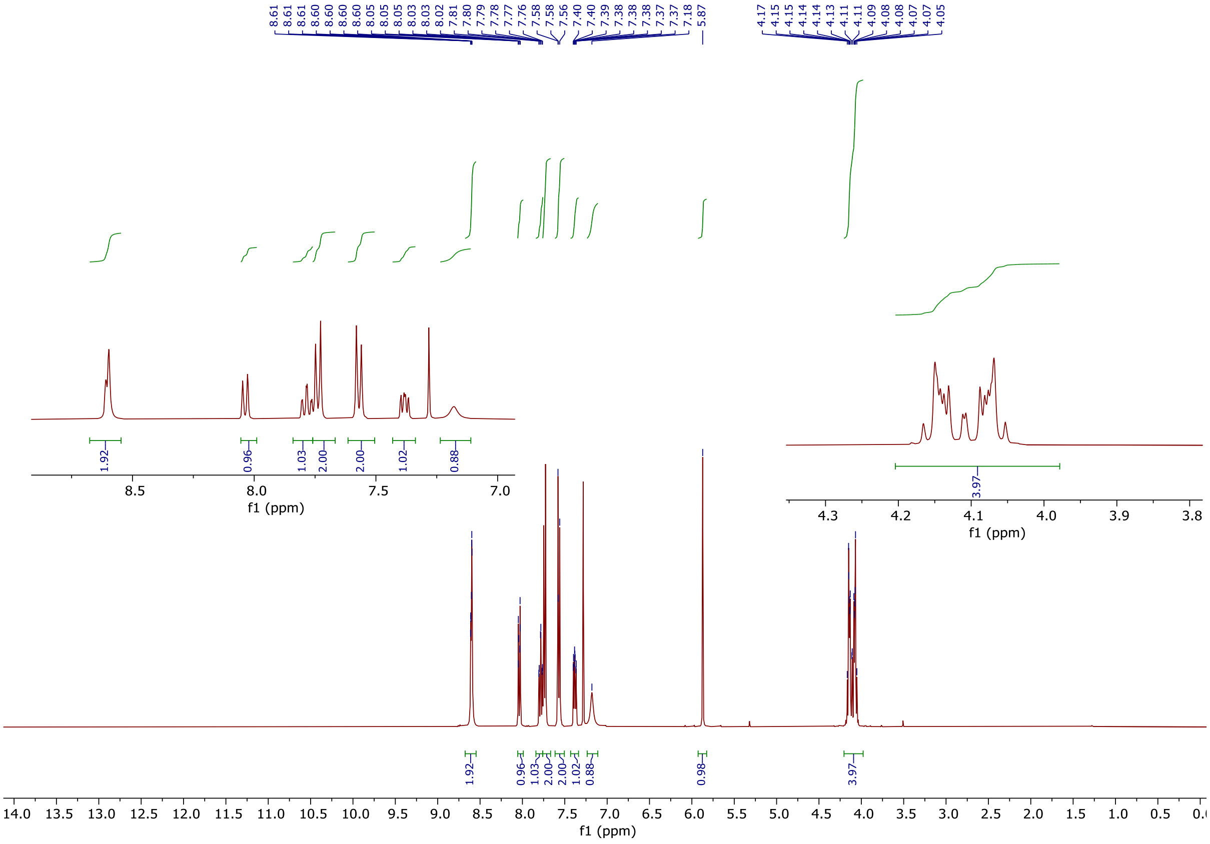

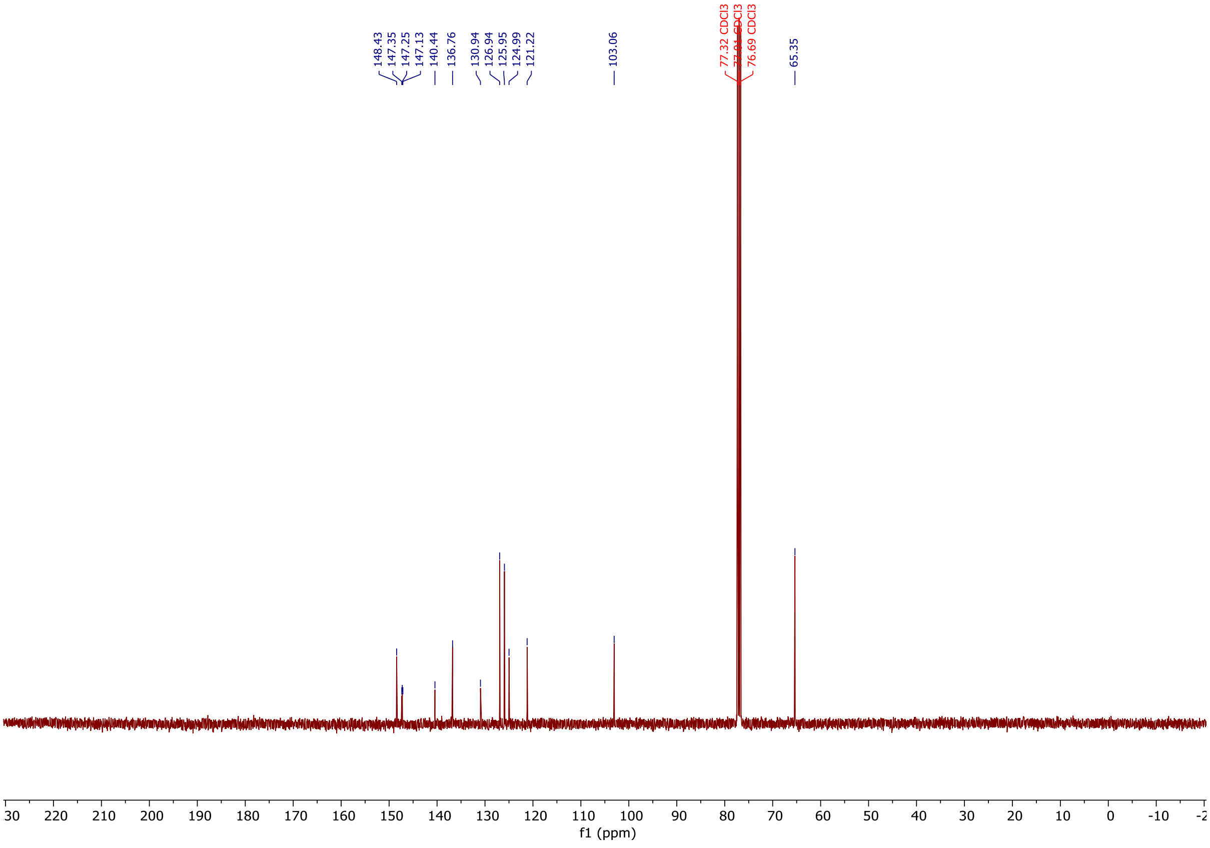

4-(6-(pyridin-2-yl)-1,4-dihydro-1,2,4,5-tetrazin-3-yl)benzaldehyde (12)

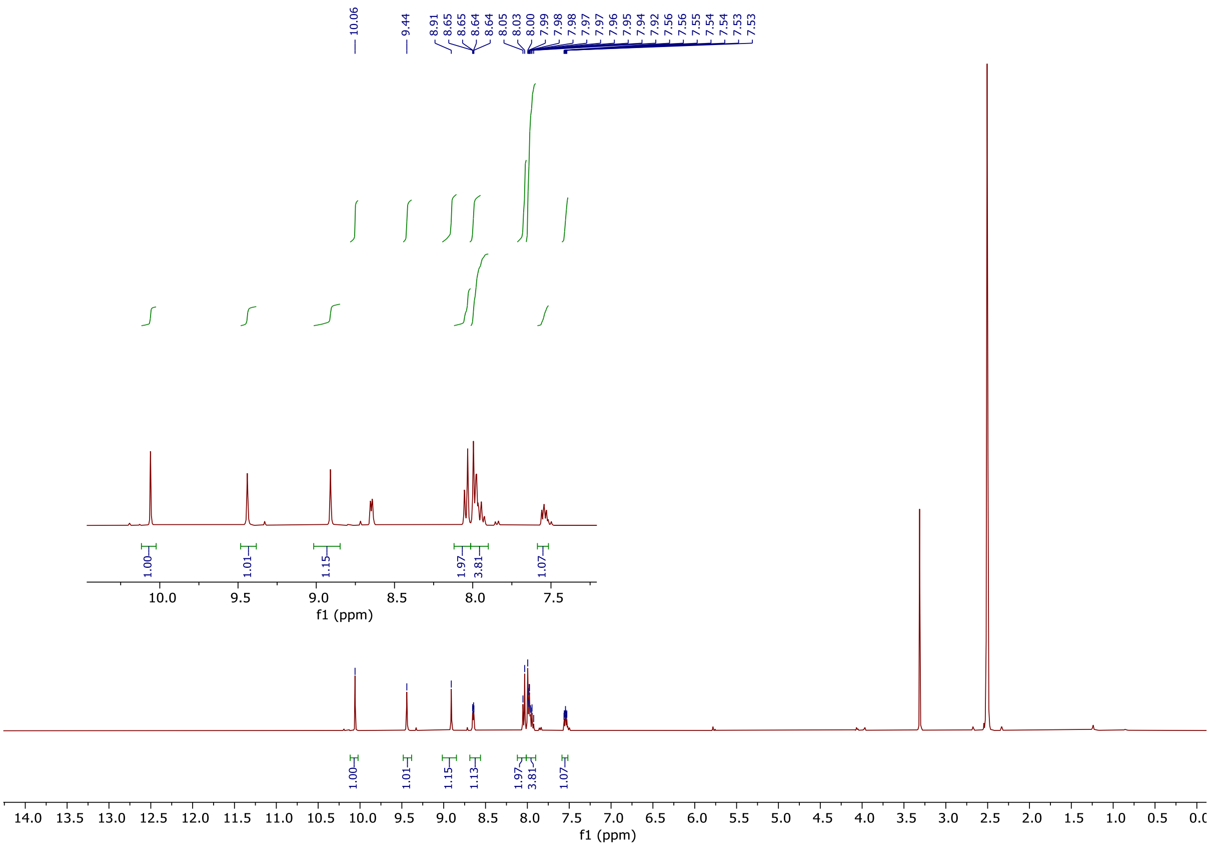

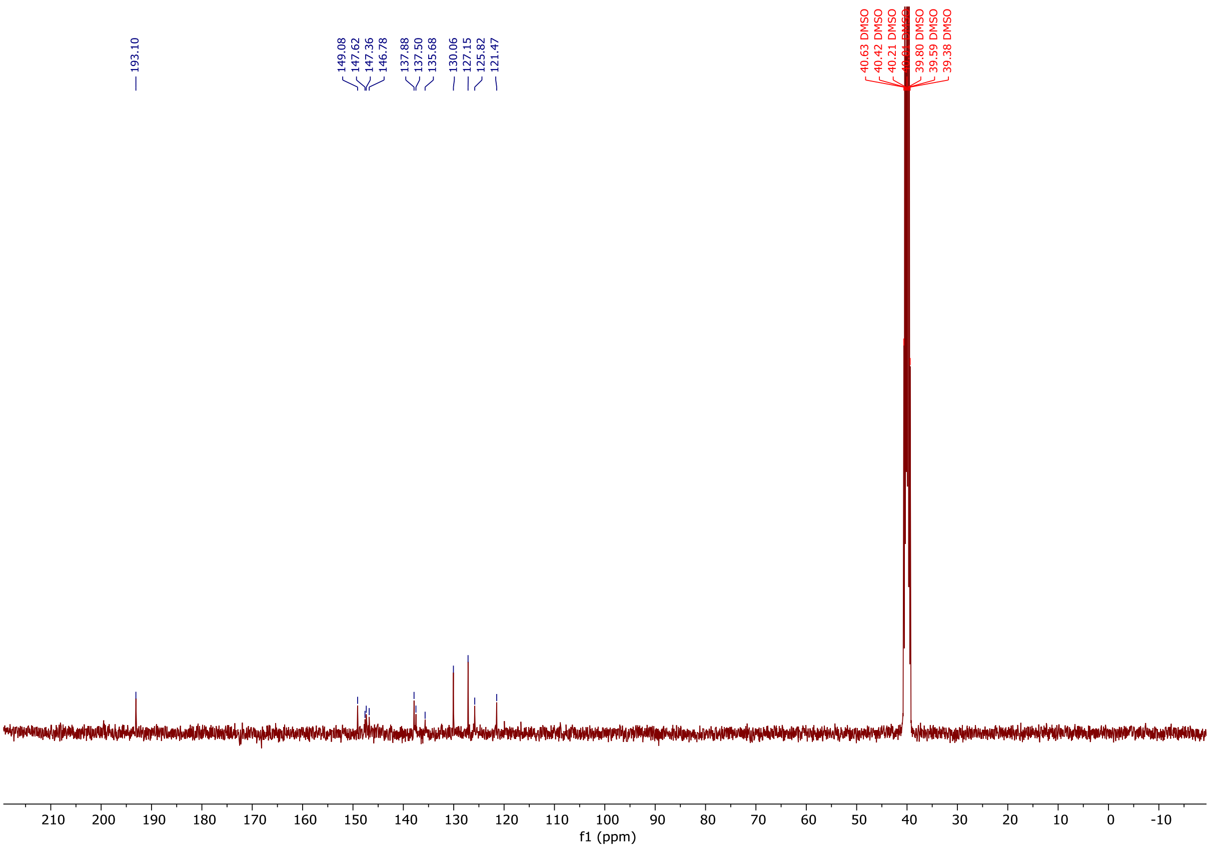

(4-(6-(pyridin-2-yl)-1,2,4,5-tetrazin-3-yl)phenyl)methanol (13)

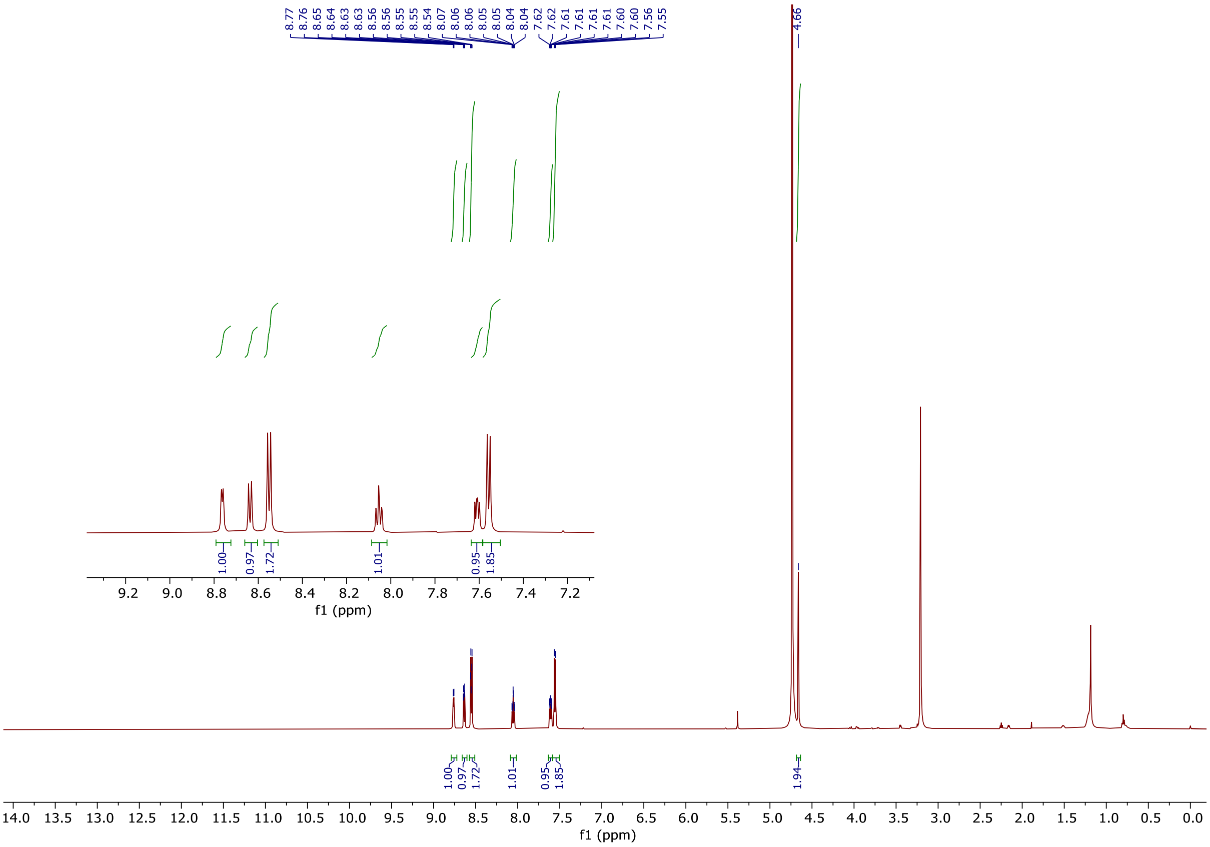

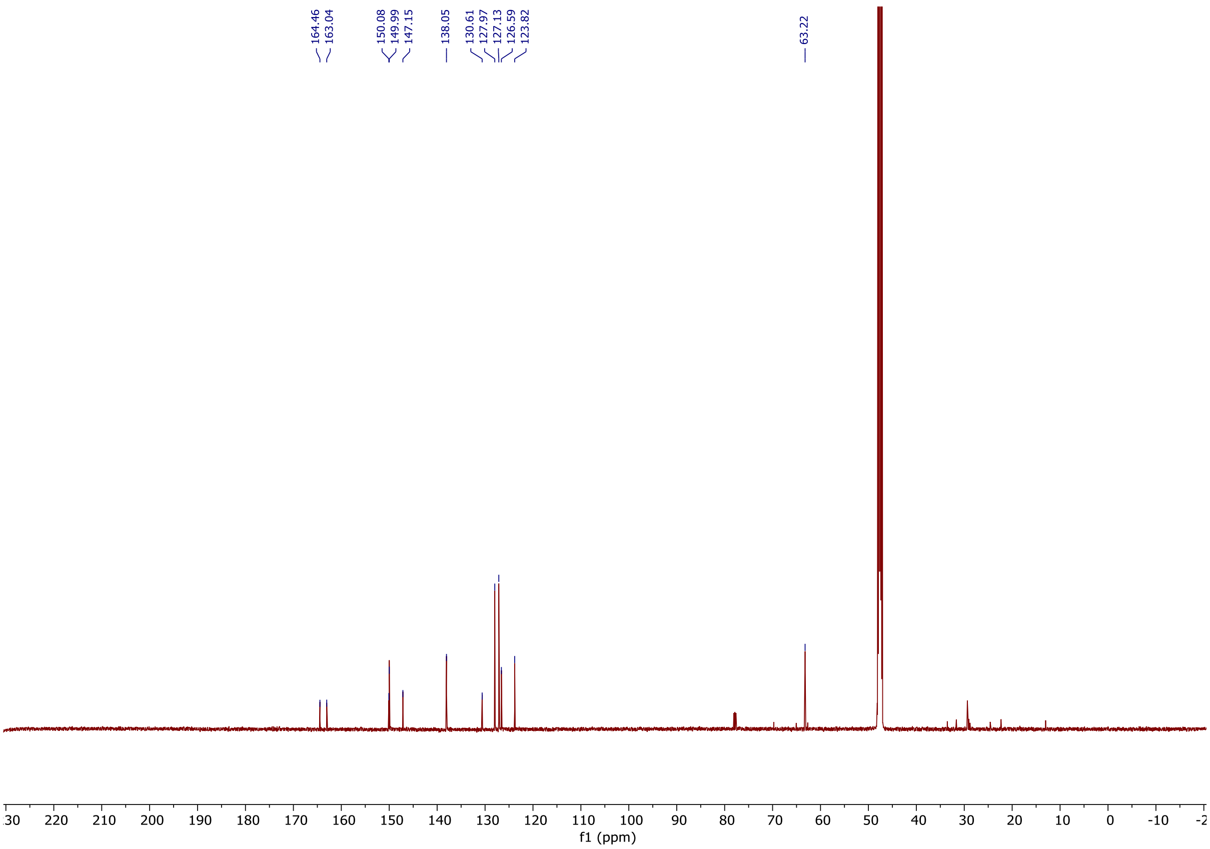

(4-(6-(Pyridin-2-yl)-1,2,4,5-tetrazin-3-yl)phenyl)methan-d-ol (14)

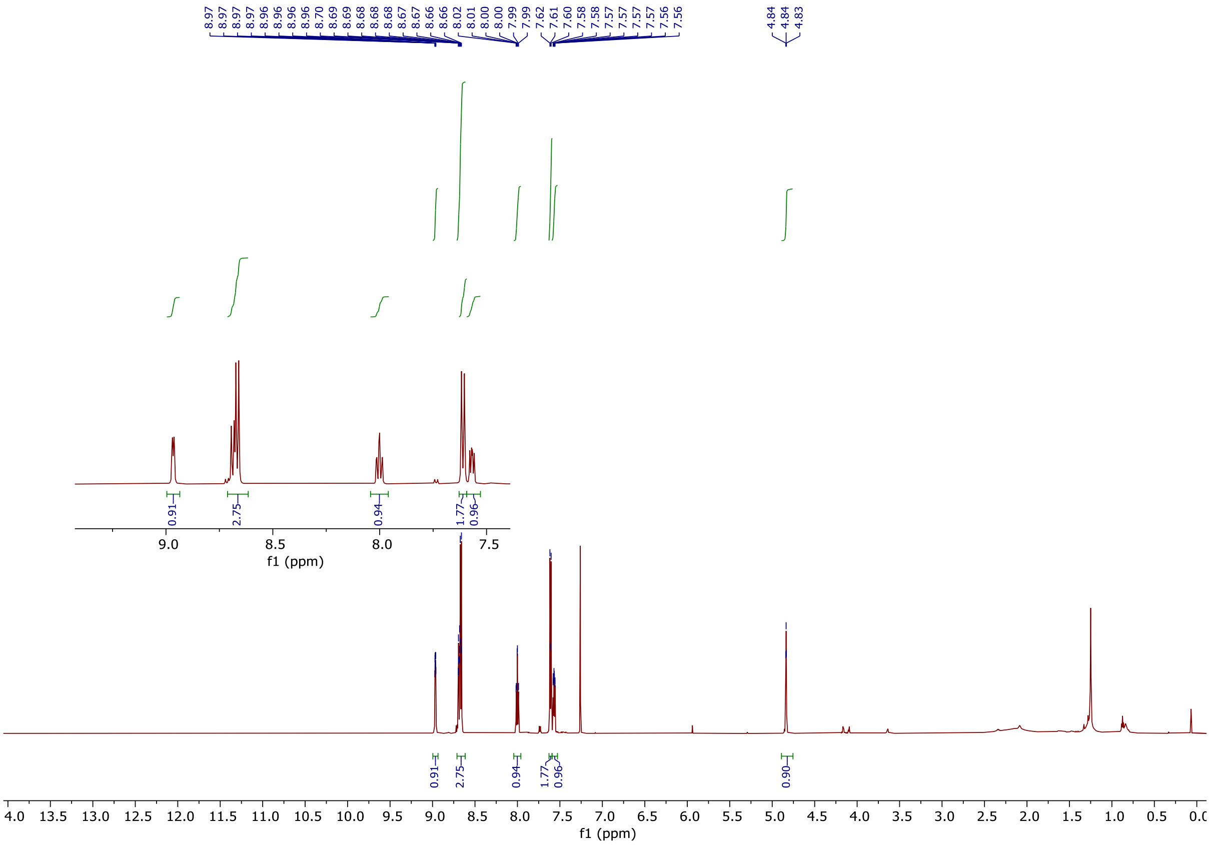

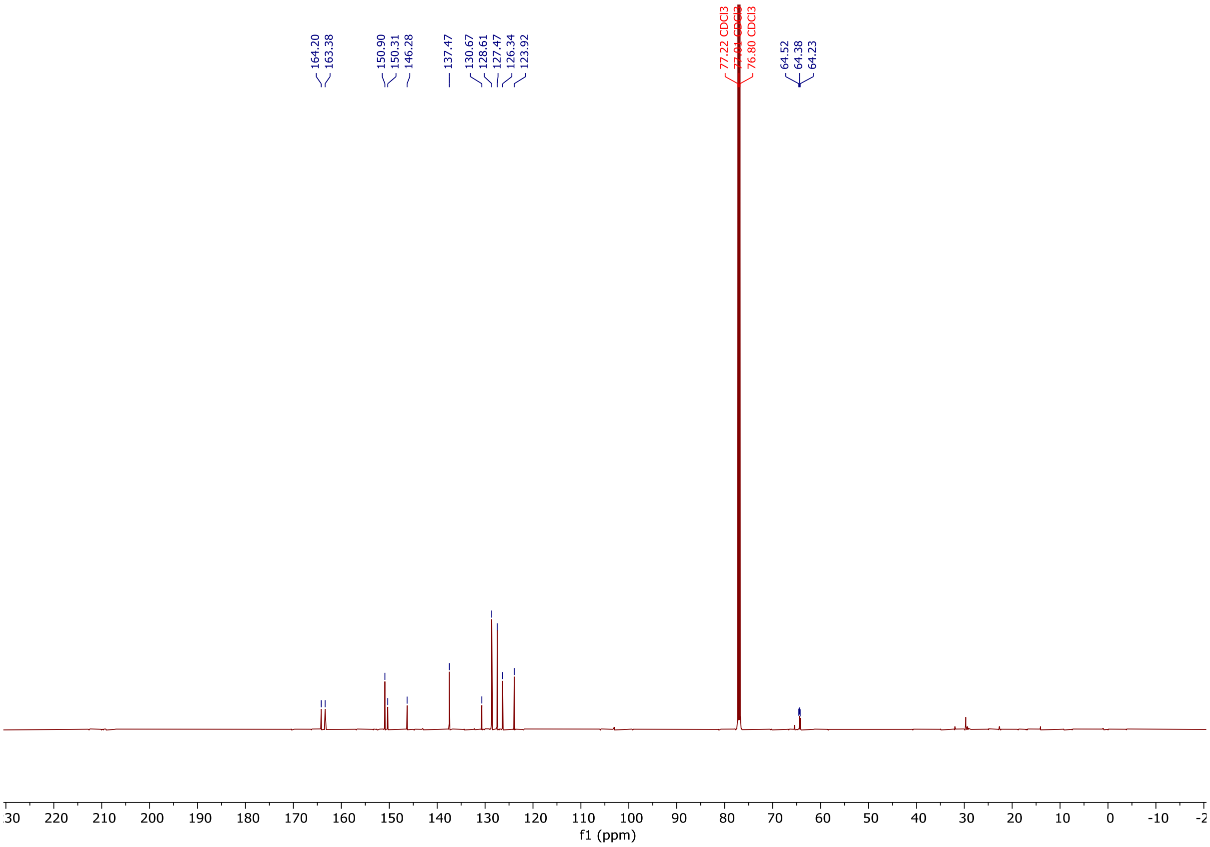

4-(6-(pyridin-2-yl)-1,2,4,5-tetrazin-3-yl)benzaldehyde (15)

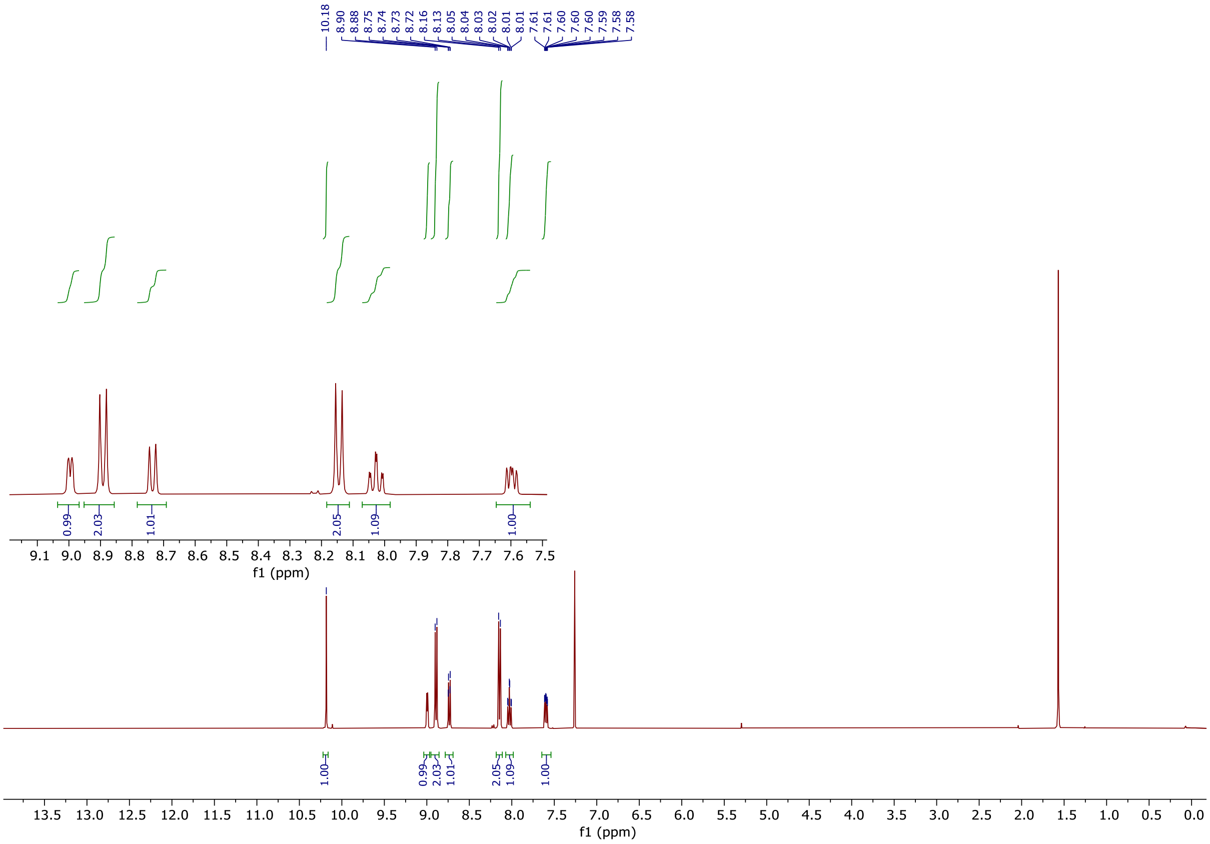

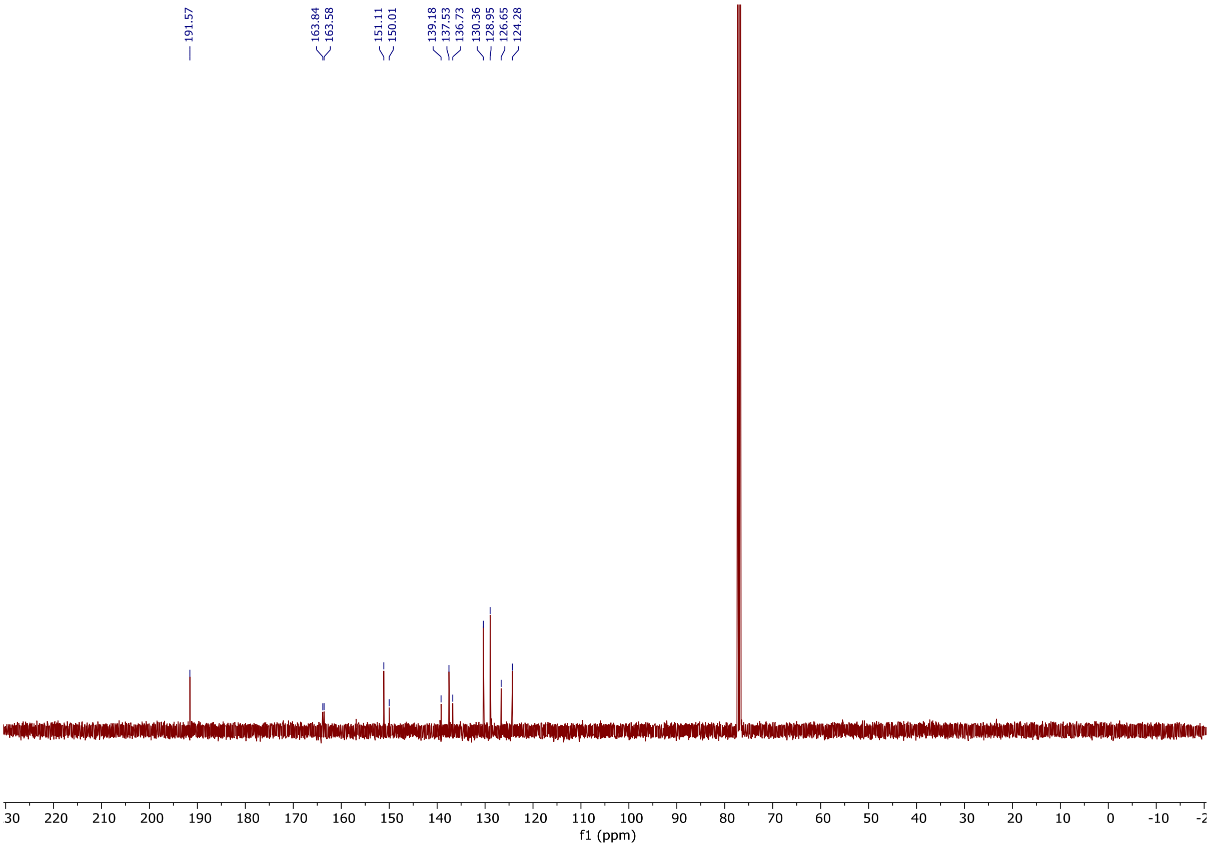

References

[1] a) in *Introduction*, **2009**, pp. 1-23; b) A. Hadj-Hamdri, A. Vidal-Cros, M. Gaudry, F. Sobrio and B. Rousseau, *Journal of Labelled Compounds and Radiopharmaceuticals* **1995**, *36*, 795-799.

[2] M. Saljoughian, H. Morimoto, P. G. Williams, C. Than and S. J. Seligman, *The Journal of Organic Chemistry* **1996**, *61*, 9625-9628.

[3] N. Ahmed and J. E. Van Lier, *Journal of Chemical Research* **2006**, *2006*, 584-585.

[4] a) G. D. Angel and J. L. Benitez, *Journal of Molecular Catalysis A: Chemical* **2001**, *165*, 9-13; b) J. B. Hoke, G. A. Gramiccioni and E. N. Balko, *Applied Catalysis B: Environmental* **1992**, *1*, 285-296.

[5] a) R. Schwob and J. Wuersch, *Journal of Labelled Compounds and Radiopharmaceuticals* **1978**, *14*, 355-360; b) M. Saljoughian, H. Morimoto and P. G. Williams, *Journal of the Chemical Society, Perkin Transactions 1* **1990**, 1803-1808.

[6] N. H. Sagert and R. M. L. Pouteau, *Canadian Journal of Chemistry* **1974**, *52*, 2960-2967.

[7] M. S. Viciu, G. A. Grasa and S. P. Nolan, *Organometallics* **2001**, *20*, 3607-3612.

[8] A. Zask and P. Helquist, *The Journal of Organic Chemistry* **1978**, *43*, 1619-1620.

[9] A. Bhattacharjya, P. Klumphu and B. H. Lipshutz, *Organic Letters* **2015**, *17*, 1122-1125.
